# Active site-targeted covalent irreversible inhibitors of USP7 impair the functions of Foxp3+ T-regulatory cells by promoting ubiquitination of Tip60

**DOI:** 10.1371/journal.pone.0189744

**Published:** 2017-12-13

**Authors:** Feng Wang, Liqing Wang, Jian Wu, Ivan Sokirniy, Phuong Nguyen, Thomas Bregnard, Joseph Weinstock, Michael Mattern, Irina Bezsonova, Wayne W. Hancock, Suresh Kumar

**Affiliations:** 1 Progenra Inc, Malvern, Pennsylvania, United States of America; 2 Division of Transplant Immunology, Department of Pathology and Laboratory Medicine, Children’s Hospital of Philadelphia and Perelman School of Medicine, University of Pennsylvania, Philadelphia, Pennsylvania, United States of America; 3 Department of Molecular Biology and Biophysics, UCONN Health, Farmington, Connecticut, United States of America; CHA University, REPUBLIC OF KOREA

## Abstract

Accumulation of Foxp3+ T-regulatory (Treg) cells in the tumor microenvironment is associated with tumor immune evasion and poor patient outcome in the case of many solid tumors. Current therapeutic strategies for blocking Treg functions are not Treg-specific, and display only modest and transient efficacy. Recent studies revealed that ubiquitin-specific protease 7 (USP7) is essential for Treg functions by stabilizing expression of Tip60 and Foxp3, which together are central to the development and maintenance of the Treg cell lineage. Pharmacological inhibition of USP7 is therefore a promising strategy for suppressing Treg functions and promoting anti-tumor immunity. Previously, we reported the P5091 series of small molecule USP7 inhibitors and demonstrated their direct anti-tumor activity *in vivo* using xenograft models. However, the precise mechanism of action of these compounds was not well defined. In this study, we report the development and characterization of P217564, a second-generation USP7 inhibitor with improved potency and selectivity. P217564 selectively targets the catalytic cleft of USP7 and modifies its active site cysteine (C223) by forming a covalent adduct. Irreversible inhibition of USP7 results in durable downstream biological responses in cells, including down-regulation of Tip60 and consequent impairment of Treg suppressive function. In addition, we demonstrate that both USP7 and various USP7 substrates are subjected to Lys48-mediated ubiquitin modification, consistent with increased proteasomal degradation of these proteins because of USP7 inhibition.

## Introduction

Foxp3^+^ T-regulatory (Treg) cells play important roles in maintaining the immune system by moderating the intensity of immune responses and preventing autoimmunity [1, 2]. The accumulation of Treg cells at the tumor site and/or in draining lymph nodes facilitates tumor immune evasion, and is associated with a negative prognosis for many solid tumors, including breast, colorectal, ovarian and non-small cell lung cancers [3–5]. Stable expression and activity of Foxp3 is essential to the development and maintenance of functional Treg cells [6], and Foxp3-mutant Scurfy mice experience lethal autoimmunity [7], as do humans with Foxp3 mutations, unless treated. By contrast, over-expression of the murine Foxp3 gene leads to hypocellular lymphoid tissues with diminished numbers of T cells and a hypoactive immune state [8]. Hence, control of Foxp3 levels and activity within a certain range is required for optimal immune functions. Current therapeutic strategies aimed at blocking Treg functions, development, or recruitment are not Treg-specific and show only modest and transient efficacy, presumably due to their co-targeting of activated T effector cells [9–11]. Thus, further efforts are required to identify novel therapeutics with the ability to modulate Treg functions selectively.

Ubiquitination is a regulatory post-translational protein modification that plays important roles in most, if not all, cellular pathways. The conjugation of ubiquitin regulates the intracellular activity of target proteins by altering their stability, localization, and/or activity. As with other post-translational modifications, such as phosphorylation and acetylation, the conjugation of ubiquitin is dynamic and can be reversed by deubiquitinating enzymes (DUBs), also known as isopeptidases. Given the critical role of the ubiquitin pathway in regulating cellular processes, targeting this pathway has the potential to treat a broad range of devastating diseases including cancer and neurodegeneration [12–15]. Recent studies revealed that Foxp3, the Treg lineage specific transcription factor and Tip60, the histone acetyltransferase that modulates Foxp3 functions are substrates of ubiquitin-specific protease 7 (USP7) [16–19]. USP7 knockdown decreases Treg-cell-mediated suppression of T effector cells both *in vitro* and *in vivo* [16]. Conditional deletion of USP7 from Tregs causes impairment of Treg function leading to lethal autoimmunity [20]. Therefore, therapeutic inhibition of USP7 is a promising strategy to suppress Treg function and unleash immune activity against tumors.

In previous work, we identified P5091, a selective inhibitor of USP7, through high throughput screening (HTS) [21–23]. P5091 and its derivative P22077 have been used widely to study the biological roles of USP7, and shown to induce degradation of many USP7 substrates and phenocopy USP7 knockdown [18, 21, 23–26]. Here, we report characterization of the mechanism of action of a more potent, second generation inhibitor, P217564, which has been shown to impair Treg functions and prevent tumor growth in syngeneic mouse models [20]. P217564 selectively binds to the active site of USP7 resulting in covalent modification of the active site cysteine. Sustained irreversible inhibition of USP7 activity by P217564 in the cell leads to the durable cellular efficacy. P217564 downregulates Foxp3 and Tip60 in Treg cells and impairs Treg functions. In addition, utilizing the UbiTest analysis we demonstrate that inhibition of USP7 increases polyubiquitination of Foxp3, Tip60 and USP7. These studies further support the feasibility of modulating Treg functions *via* pharmacological inhibition of USP7. In addition, the assays described in this work will have considerable utility in preclinical and clinical evaluation of USP7 inhibitors.

## Methods and materials

### Protein production and purification

Recombinant full-length USP5, USP7, and SENP2 catalytic core, as well as Ub-EK_L_ (Ub-CHOP2) and SUMO1-EKL were generated as previously described [27, 28]. EK_L_ was generated by cleaving SUMO3- EK_L_ with SENP2 core, and mature EK_L_ was purified by affinity chromatography. The wild type and catalytic mutant (active site cysteine 223 replaced by alanine, C223A) USP7 core proteins were produced in *E*.*coli* and purified through a His-tag, with nickel affinity chromatography. A plasmid used for production of isotopically labeled USP7 catalytic domain for NMR experiments was a gift from Structural Genomics Consortium (residues 208–560 in pET28a-LIC vector). The ^2^H/^15^N-labeled domain was expressed in *E*. *coli* BL21 (DE3) strain using 100% D_2_O-based M9 minimal medium supplemented with ^15^NH_4_Cl and ^12^C/^2^H -glucose as the sole source for nitrogen and carbon, respectively. Protein expression was induced at an OD_600_ ~ 0.8 by adding 1 mM IPTG. Cells were harvested after 16 hours of incubation at 20°C and lysed by sonication in extraction buffer containing 20 mM NaH_2_PO_4_, 250 mM NaCl, 10 mM imidazole, pH 7.4. Cell lysates were clarified by centrifugation at 15000 rpm for 45 min and applied to a Ni-NTA resin (Thermo Scientific). Recombinant protein was eluted with extraction buffer containing 250 mM imidazole. Following thrombin digestion to remove the His_6_-tag, catalytic domain was additionally purified by size exclusion chromatography using HiLoad Superdex 200 column (GE Healthcare) in buffer containing 20 mM Tris-HCl, 100 mM NaCl, 2 mM DTT, pH 7.5. All NMR samples additionally contained 10% D_2_O.

### Cell culture

Human colorectal cancer HCT116 (p53^WT^) and human prostate cancer PC-3 (p53^MUT^) cell lines (obtained from ATCC) were maintained in Dulbecco's Modified Eagle Medium (DMEM) supplemented with 10% FBS (Biowest), 2 mM L-glutamine (GIBCO), and 50 units/mL penicillin/streptomycin (GIBCO). Human T lymphocyte cell line Jurkat (p53^MUT^) was maintained in Roswell Park Memorial Institute (RPMI) 1640 Medium supplemented with 10% FBS, 2 mM L-glutamine, and 50 units/mL penicillin/streptomycin.

### Cysteine protease reporter assay

The ability of P217564 to inhibit a panel of deubiquitinases including USP2 (2 nM), USP5 (40 nM), USP7 (4 nM), USP8 (8 nM), USP15 (8 nM), USP20 (2 nM), USP21 (5 nM), USP28 (40 nM), and USP47 (0.25 nM) was tested using reporter substrate Ub-EK_L_ (10 nM) and QXL (20 nM). P217564 was also tested for inhibition of the catalytic activities of SENP1 (0.5 nM), Calpain 1 (250 nM, Millipore, 208712), Caspase 3 (0.5 nM, R&D Systems, 707-C3), Cathepsin K (0.1 nM, Millipore, 219461-25UG) and MMP13 (312.5 pM, Anaspec, 72011), using the SUMO1-EKL (10 nM), calpain fluorogenic substrate 1 (5 μM, Millipore, 208748), DEZD-Rh110 (250 nM, Anaspec, AS-60304-5), PICL-10 (10 μM), and 520 MMP FRET substrate 1 (250 nM, Anaspec, 60568–01) substrates, respectively. All enzymes were incubated with DMSO or compounds for 60 min at room temperature before initiation of the enzymatic reaction by addition of substrates. The reactions were monitored using the Perkin Elmer Envision model number 2101.

### Ub-VME based USP7 activity assay

Compound- or DMSO-treated cells were washed once with PBS and then harvested and lysed in 1% NP40 lysis buffer (50 mM Tris-HCl, pH 7.5, 150 mM NaCl, 1% NP40, 10% Glycerol, 1 mM PMSF, 2 mM β-ME). 20 μg of lysate per sample was used for the Ub-VME assay by incubating with Ub-VME at a final concentration of 400 nM. Total reaction volume was 10 μl. The reaction was performed at 37°C for 30 min and then stopped by adding 2 μl of 6X SDS sample buffer and boiling for 5 min, followed by SDS-PAGE electrophoresis, transfer to PVDF membranes, and blotting with USP7-specific antibody to determine the formation of USP7-Ub-VME complex.

### Western blots

Following compound treatment at the indicated time and concentrations, cells were washed once with PBS, harvested and lysed in RIPA lysis buffer (50 mM Tris-HCl, pH 7.5, 150 mM NaCl, 1% NP40, 1% Sodium deoxycholate, 2 mM EDTA, 10% Glycerol, Protease inhibitor cocktail (Sigma cat #P8849, 1:500), 50μM PR-619 (pan-DUB inhibitor, LifeSensors Cat#SI9619)). Proteins were quantified using Bradford’s method (Bio-Rad) following the manufacturer’s instructions. 25 μg of cell lysates were separated on 10% SDS-PAGE gel, transferred to PVDF membranes and probed with antibodies against PARPc (Cell Signaling, 9541), HDM2 (Santa Cruz, sc-965), UHRF1 (Santa Cruz, sc-166898), p21 (Cell Signaling, 2946), USP7 (Progenra), Actin (Sigma, A2228), Tip60 (Millipore, 07–038), Foxp3 (eBioscience, 13-5773-82), ubiquitin (Lifesensors, Vu101), and HA-tag (Covance, MMS-101P). Horseradish peroxidase (HRP)-conjugated anti-mouse (Jackson Lab) or HRP-conjugated anti-rabbit (Jackson Lab) were used as secondary antibodies. Blots were developed using Millipore ECL or Pierce ECL depending on the sensitivity requirements.

### Immunoprecipitation

USP7 immunoprecipitation and activity measurement were performed as reported previously [21], with slight modifications. Briefly, Jurkat cells treated with DMSO or USP7 inhibitor were washed with ice cold PBS and lysed using NP40 lysis buffer (50 mM Tris-HCl, pH 7.5, 150 mM NaCl, 1% (v/v) NP40, 10% (v/v) Glycerol, 1 mM PMSF) and pre-cleared using 30 μl of Protein G-sepharose (Invitrogen) for 1 hr at 4°C. 500 μg of pre-cleared cell lysates were incubated with Protein G-sepharose beads pre-loaded with 0.5 μg of anti-USP7 antibody (Progenra) for 1–2 hours. Beads were washed three times with lysis buffer containing 2 mM β-mercaptoethanol and USP7 activity was determined using Ub-EK_L_ and EK_L_ substrate as described above. DMSO treated cell lysates were incubated with beads alone for control. 5 μl of beads were analyzed by immunoblotting with anti-USP7 antibody to determine relative levels of USP7.

### Quantitative RT-PCR

Quantitative RT-PCR was carried out as described previously [20]. Briefly, total RNA from murine Foxp3+ Tregs and HCT116 cells treated with DMSO or P217564 for indicated time points was isolated using RNeasy Kits (Qiagen). cDNA was synthesized with TaqMan^®^ Reverse Transcription Reagents (Applied Biosystems) and quantitative PCR was performed using specific primers obtained from Applied Biosystems (Catalogue Numbers: Human Tip60; Hs00197310_m1, Human p53; Hs01034249_m1, Human Mdm2; Hs00540450_s1, mouse Tip60; Mm01231512_m1 and mouse Foxp3; Mm00475162_m1; Eukaryotic 18S rRNA; 4319413E) and TaqMan Universal PCR Master Mix (Applied Biosystems). Gene expression data was normalized to 18S rRNA.

### Annexin V / Propidium Iodide (PI) apoptosis assay

Jurkat cells were pre-treated with DMSO, 1 μM, or 5 μM P217564 for 4 hours or 16 hours, washed twice with cold PBS and resuspended in 1 x Binding Buffer (BD Biosciences, 556454) at a concentration of 1 x 10^6^ cells/mL. 100 μL of cells (1 x 10^5^ cells) were transferred to a new tube and incubated with 5 μL of FITC Annexin V (BD Biosciences, 556419) and 10 μL of PI (BD Biosciences, 556463) for 15 minutes at room temperature in dark. Samples were diluted 5 times with 1 x Binding Buffer and analyzed by flow cytometry (BD Accuri C6) within 1 hour.

### DUB-based UbiTest assay

The UbiTest kit was purchased from Lifesensors (UM411), and the assay was performed following the manufacturer’s instructions. Briefly, PBS washed cells were lysed in cell lysis buffer (50 mM Tris-HCl, pH 7.5, 0.15M NaCl, 1mM EDTA, 1% NP-40, 10% glycerol, RIPA (ThermoFisher, PI89900)). Cell lysate (500 μg per sample) was incubated with equilibrated Agarose-TUBEs at 4°C for 2 hours. Beads were washed 3 times with 1 mL TBS-T buffer, followed by elution of pull down products with elution buffers. Eluted proteins were neutralized with neutralization buffer, and then incubated with DUBs at 37°C for 0.5–1 hr. The increased signal of protein of interest post DUB treatment was detected using immunoblot analysis.

### NMR spectroscopy

All NMR experiments were recorded at 30°C on 800 MHz (^1^H) Agilent VNMRS spectrometer equipped with a cryo-probe. NMR data were processed with NMRPipe and analyzed with Sparky [29]. P217564 binding to USP7 catalytic domain was assessed by comparing ^1^H-^15^N HSQC spectra of free USP7 (208–560) and USP7 in the presence of P217564 inhibitor. A 4-molar excess of P217564 inhibitor was used. Both free and P217564-bound NMR samples contained 5% DMSO. Backbone chemical shift assignment of the catalytic domain of USP7 (BMRB ID: 29951) previously reported by us was used to identify the amide backbone resonances in ^1^H-^15^N HSQC spectra [30]. Frequency (chemical shift) perturbations were calculated for each amino acid residue as Δω_*i*_ = (Δω_N_^2^+Δω_H_^2^) ^1/2^, where Δω_N_ and Δω_H_ are ^15^N and ^1^H frequency differences between P217564-bound and free states, respectively. The obtained Δω_*i*_ values were mapped onto the surface of the crystal structure of USP7 catalytic core (PDB ID: 1NB8) [31].

### Mass spectrometry

5 μM purified USP7 core WT or C223A was incubated with 100μM P217564 or DMSO at room temperature for 4 hours, and then the protein/compound reaction products were mixed with equal volumes of 0.1% Trifluoroacetic acid (TFA) before LC-MS injection. The samples were analyzed by reverse-phase liquid chromatography coupled to UV and MS detection (Agilent, 6220 Accurate-Mass Time-of-Flight (TOF) LC/MS). For the conjugation site mapping experiment, trypsin-digested USP7/P217564 covalent complex was analyzed by high-resolution LC-MS/MS.

### Treg suppression assays

Mice were euthanized by barbiturate overdose. CD4+CD25- T-effector and CD4+CD25+ Treg cells were isolated from C57BL/6 mice using CD4+CD25+ Treg isolation kits (130-091-041, Miltenyi Biotec). Teff cells (5x10^5^) were labeled with Cell Trace Violet and stimulated with CD3 mAb (5 μg/ml) in the presence of 5 × 10^5^ irradiated syngeneic T-cell depleted splenocytes (130-049-101, Miltenyi Biotec) and varying ratios of Tregs [32]. After 3 days, proliferation of Teff cells was determined by analysis of Cell Trace Violet dilution [20].

## Results

### P217564 is a second generation USP7 inhibitor with increased potency

**Fig 1:**
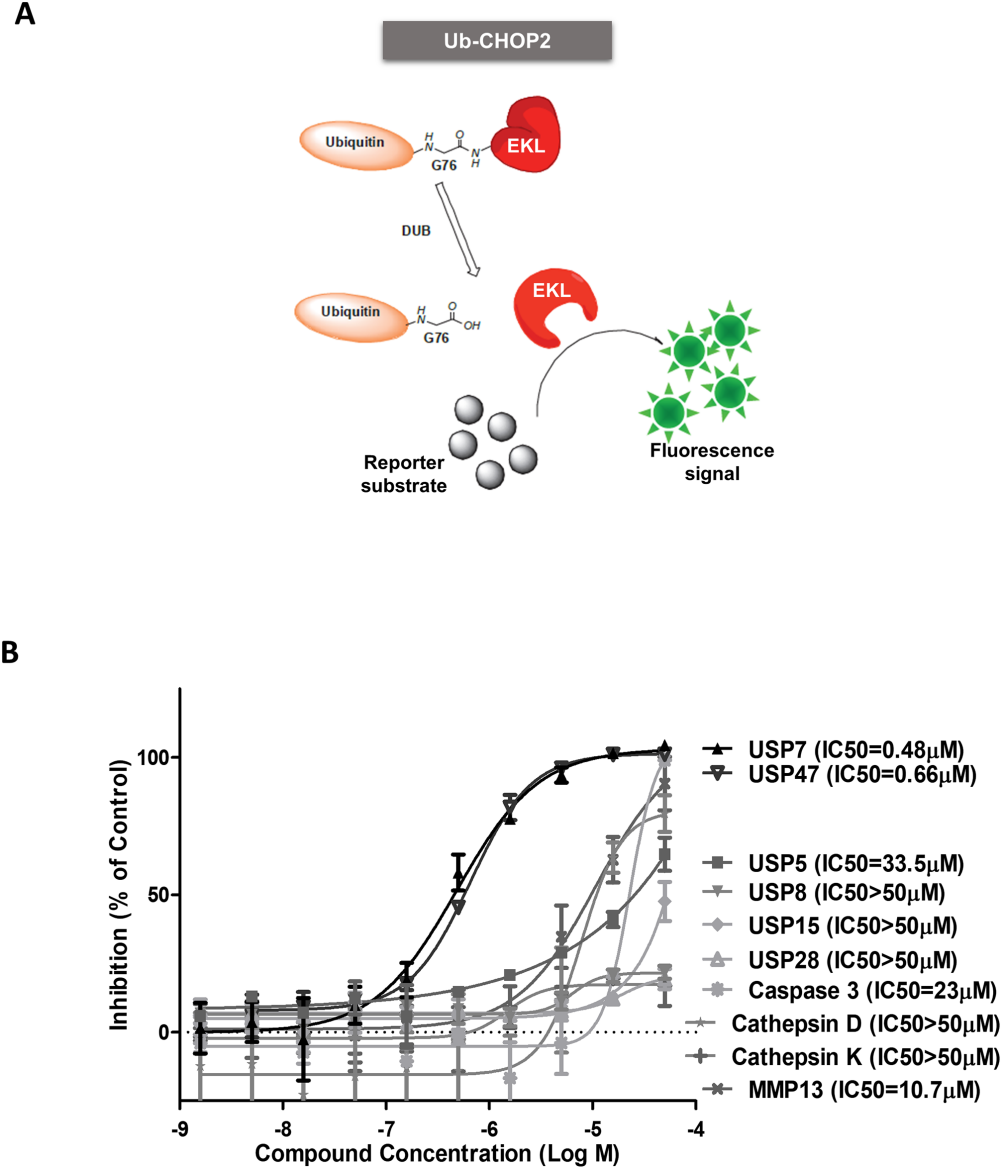
P217564 is a second generation USP7 inhibitor with increased potency that retains selectivity. (A) Schematic representation of Ub- EK_L_ isopeptidase reporter assay: cleavage by the isopeptidase at the carboxy-terminal glycine of the Ub releases catalytically active EK_L_, which liberates a quantifiable fluorescent product in the presence of its substrate. (B) P217564 demonstrates potent and specific inhibition of USP7 versus other purified DUBs and proteases.

Using the previously identified and selective USP7 inhibitor, P5091 (IC_50_ = 4.2 μM, [21]), as the starting point, we synthesized a series of compounds to optimize potency and drug-like properties [22]. Inhibition of USP7 activity was evaluated using the Ub-EK_L_ (Ub-CHOP2) reporter assay [27], in which ubiquitin is fused to the reporter enzyme enterokinase light chain (EK_L_), rendering it catalytically inactive. Following cleavage of the Ub-reporter fusion by the isopeptidase, the liberated EK_L_ subsequently acts on its substrate, generating a signal that is linear with deubiquitylase (DUB) concentration, allowing a quantitative measure of DUB activity (Fig 1A) [27]. P217564 inhibited USP7 with a potency of 0.48μM, indicating a ~10-fold increase in potency compared to P5091 (EC50 = 0.48 μM vs 4.2 μM (Fig 1B)). To evaluate the selectivity of P217564, we compared its inhibitory potency against the potencies of a representative set of purified recombinant isopeptidases. Notably, none of the tested USPs were inhibited significantly, except USP47, the closest structural homologue of USP7, which was inhibited with a potency similar to that of USP7 (Fig 1B). We also tested the inhibitory effect of P217564 on other proteases, including Caspase3, MMP13, Cathepsin D, and Cathepsin K. As shown in Fig 1B, P217564 inhibited USP7 with ≥ 20-fold higher potency as compared with its inhibition of the other proteases.

**Fig 2:**
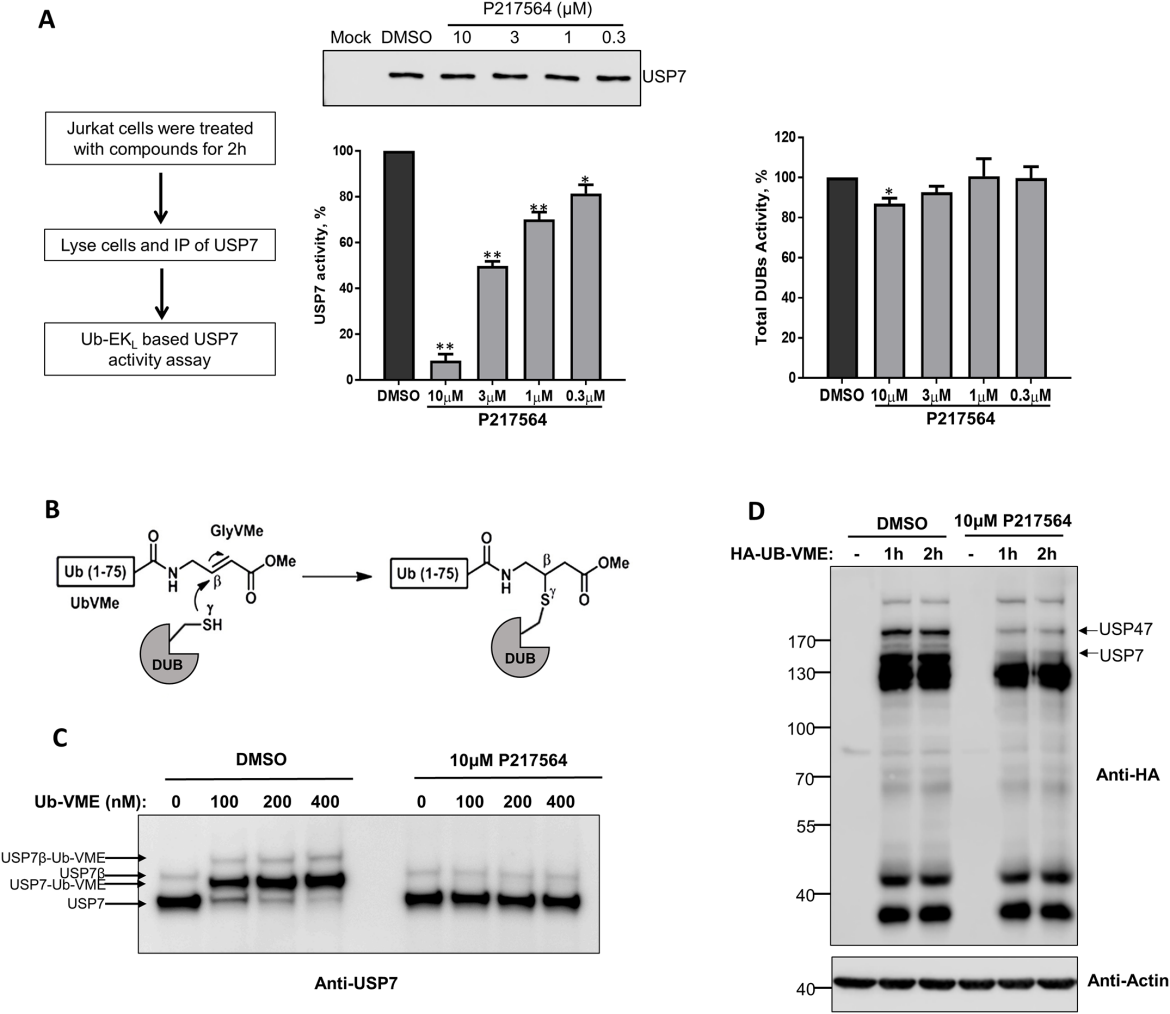
P217564 selectively inhibits USP7 activity in cultured cells. (A) Jurkat cells were treated with DMSO or P217564 as indicated for 2 hours; USP7 was isolated by immunoprecipitation (IP) and its activity quantified by UB-EK_L_-based activity assay (middle). Simultaneously, equal amounts of crude cell extracts were used to determine total DUB activity using the UB-EK_L_ assay (Right). (B) Schematic of Ub-VME reaction. (C) Jurkat cells were treated with DMSO or P217564; crude cell lysates were incubated with the Ub-VME probe and then immunoblotted against USP7 to determine formation of USP7-Ub-VME complexes. (D) Jurkat cells were treated with DMSO or P217564; crude cell extracts were labeled with HA-Ub-VME probe, followed by immunoblotting (IB) with indicated antibodies.

To confirm the cellular potency and selectivity of P217564, we performed a cell-based dose-response assay, in which Jurkat cells were pretreated with serially diluted compound (0.3 to 10 μM for 2 hours. Compound-treated cells were then washed in ice-cold PBS, harvested, lysed and equal amounts of cell lysates subjected to anti-USP7 immunoprecipitation. The activity of isolated USP7 was evaluated using the Ub-EK_L_ assay. P217564 inhibited cellular USP7 in a dose-dependent manner, with cellular IC_50_ ~3 μM (Fig 2A). The Western blot result showed that the amounts of isolated USP7 were roughly equal, indicating that the observed decrease in USP7 activity was not due to a decrease in USP7 content. The total cellular DUB activity was also quantified by assaying crude cell lysate in the Ub-EK_L_ assay. Total DUB activity was minimally affected, even at a compound concentration of 10 μM, which inhibits > 90% activity of USP7, confirming the cellular selectivity of compound P217564.

We also employed a second approach to determine USP7 activity using an ubiquitin vinyl methyl ester (Ub-VME) based assay. Ub-VME is a potent suicide inhibitor probe of DUBs by virtue of its binding DUBs covalently at their active site cysteine (Fig 2B). Reaction with Ub-VME leads to the formation of USP7-Ub-VME complex, resulting in an 8 kD increase in the molecular weight of USP7 protein. Because only catalytically active USP7 can react with Ub-VME, the level of USP7-Ub-VME complex formation represents the activity of USP7. Cell extracts obtained from DMSO, P5091 (30 μM) or P217564 (10 μM) treated cells were allowed to react with Ub-VME, and then subjected to SDS-PAGE separation and immunoblotting against USP7. Consistent with our results from the Ub-EK_L_ based assay, 10 μM P217564 as well as 30 μM P5091 inhibited nearly all of the activity of cellular USP7, thereby blocking formation of USP7-Ub-VME complex (Fig 2C, S1A Fig).

In addition to analyzing the activity of a given DUB through western blotting with an antibody specific for that DUB, the Ub-VME based assay can be used to evaluate the effect of an inhibitor against numerous DUBs in parallel, by Western blotting against the Ub-VME probe itself. For this purpose, Jurkat cells were treated with DMSO or P217564, and crude cell lysate was reacted with HA-tagged Ub-VME and then subjected to SDS-PAGE electrophoresis, transferred to PVDF membrane, and immunoblotted with anti-HA antibody. As shown in Fig 2D, the overall pattern of active cellular DUBs capable of reacting with Ub-VME probe remained largely unchanged in compound treated sample compared to DMSO treated sample. However, the intensities of bands corresponding to HA-Ub-VME-USP7 and HA-Ub-VME-USP47 (anti-USP7 and USP47 blots were shown in S1B Fig to indicate the size of HA-Ub-VME-USP7 and HA-Ub-VME-USP47) were substantially decreased in the compound-treated sample suggesting selective inhibition of these enzymes by the compound. This result is consistent with the in vitro inhibitory activity of this molecule against purified USP7 and USP47 (Fig 1C). Taken together, these results indicate that P217564 is a potent and relatively selective USP7 inhibitor.

### P217564 inhibitor targets the catalytic cleft of USP7

**Fig 3:**
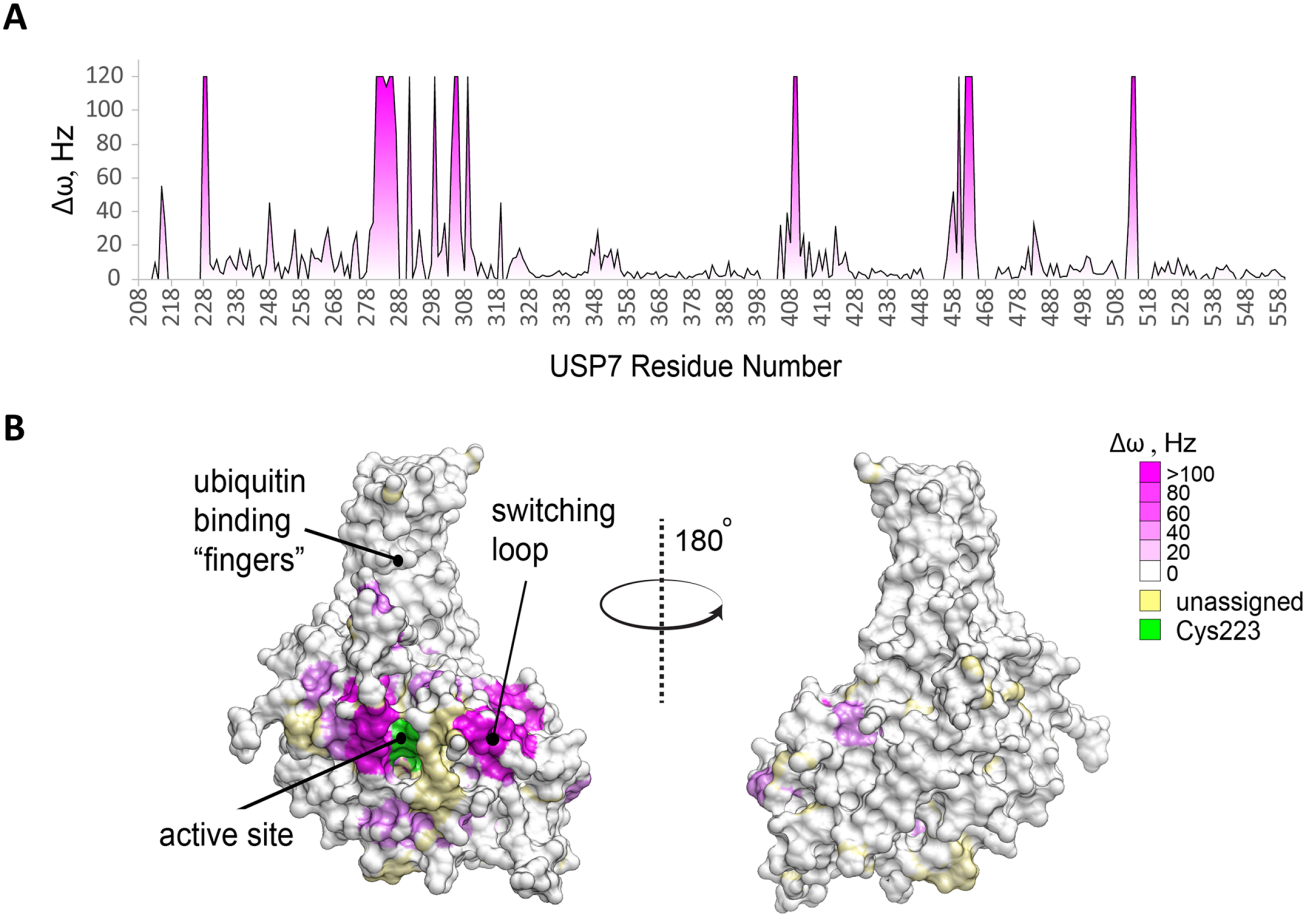
NMR chemical shift perturbation analysis of P217564 binding to USP7core and mapping of the inhibitor-binding site. (A) Inhibitor-induced chemical shift perturbations (Δω, Hz) plotted for each residue of USP7 core. Resonances that were detected in the free protein only but disappeared in the complex were arbitrarily set to 80 Hz. (B) USP7 core solvent accessible surface colored according to NMR chemical shift perturbation from (A) (pdb ID: 1NB8). The degree of magenta represents increased chemical shift changes induced by P217564 binding. USP7 core residues for which resonance assignments are missing in ^1^H-^15^N HSQC spectra are shown in light gold. Catalytic cysteine residue C223 is shown in red. The structure is shown in two orientations. The active site and the ubiquitin-binding “fingers” region are labeled.

The USP7 isolated from P217564-treated Jurkat cells by immunoprecipitation (IP) remained inhibited (Fig 2A), suggesting that the enzyme was still being engaged by compound even after IP purification. Moreover, pre-treatment of cells with P217564 prevented USP7 from reacting with Ub-VME (Fig 2C and 2D), suggesting potentially tight engagement of P217564 to USP7 protein. To identify the inhibitor binding site on the surface of USP7, NMR chemical shift perturbation assays were used. Significant chemical shift perturbations were observed in the ^1^H-^15^N HSQC spectrum of USP7core treated with P217564 compared to untreated USP7 core (S2 Fig). NMR chemical shift assignment of USP7 core backbone amide resonances previously reported by us was used to quantify per-residue changes induced by the inhibitor (Fig 3A). Per-residue chemical shift perturbations, Δω, calculated as a distance between the free and bound peaks. For several residues largely perturbed by the compounds, Δω could not be accurately estimated because their “bound” state resonances could not be unambiguously assigned. Those were arbitrary set to 80 Hz to indicate large perturbations (Fig 3A). Chemical shift perturbations of more than 20 Hz were considered significant and were mapped onto the surface of USP7 catalytic domain (PDB ID: 4M5W) (Fig 3B). As expected, the perturbations are localized around the catalytic cleft of the USP7 core and, include “switching loop” and helix 5 of the domain. Remarkably, the same structural regions undergo conformational changes during enzyme activation, when active site of the enzyme is covalently modified by ubiquitin-aldehyde [31]. These results suggest that P217564 compound targets the active site region of USP7 enzyme.

### Covalent mode of inhibition of USP7 by P217564 compound

**Fig 4:**
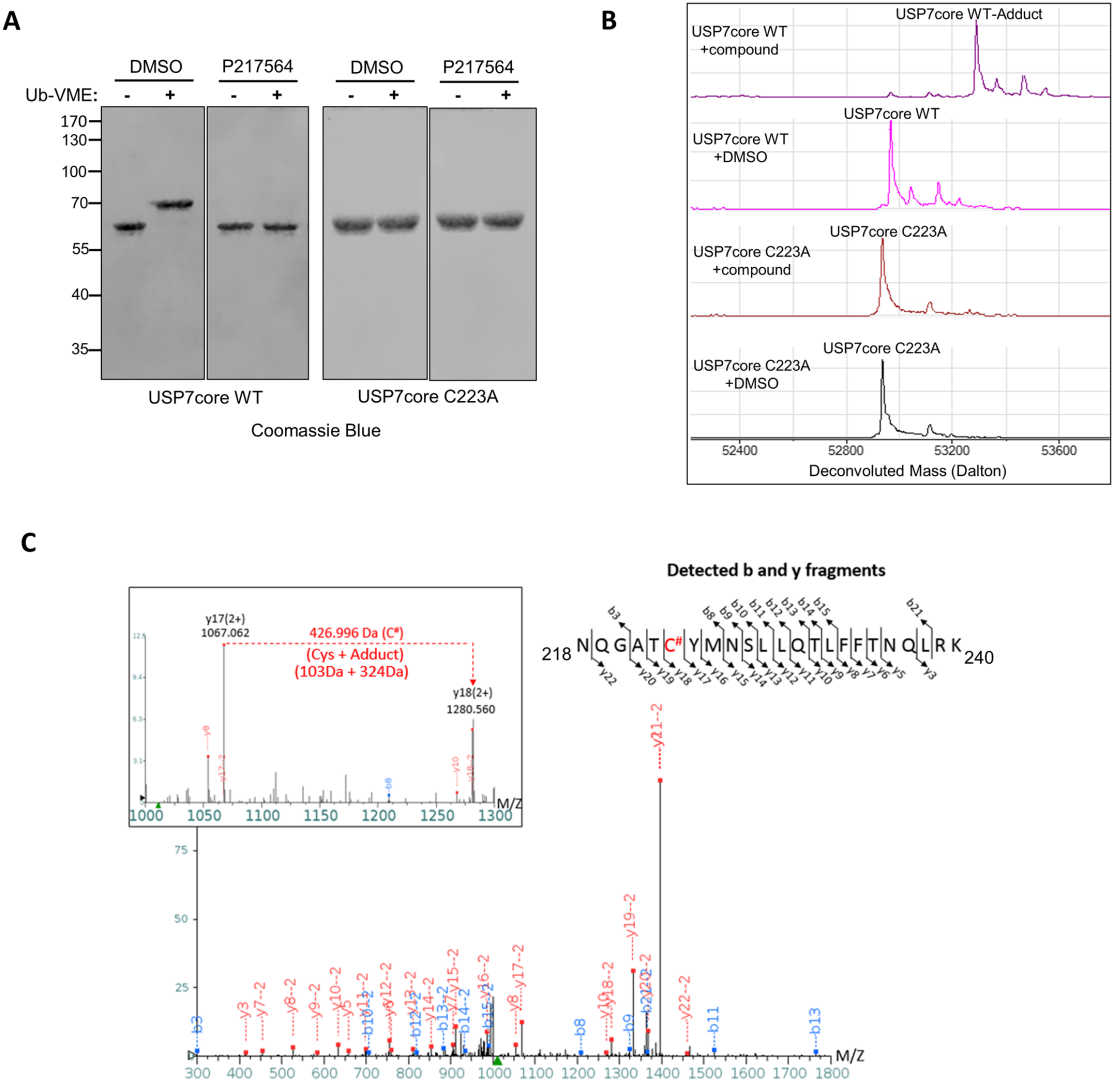
Covalent mode of inhibition of USP7 inhibitor. (A) Purified wild-type USP7 core (USP7core WT) is able to form a complex with Ub-VME; P217564 prevents the formation of the USP7 core-Ub-VME complex. Purified active site mutant USP7 core-C223A cannot react with Ub-VME. (B) P217564 binds to USP7 covalently at its active site cysteine. Purified USP7core WT or C223A was incubated with either DMSO or P217564, and then subjected to LC-MS analysis to detect the formation of compound adduct on the USP7 core protein. (C) LC-MS/MS analysis to identify the conjugation site of P217564 on USP7.

To identify the precise nature of inhibition, USP7 core was incubated with P217564 at 1:20 protein:compound ratio for 4 hours, and then subjected to LC-MS analysis. Interestingly, an increase in mass of 324 Daltons was detected after the USP7 core had been incubated with P217564 (Fig 4B), indicating the formation of a covalent complex between USP7 core and P217564. The increase in mass of the complex is less than the molecular weight of P217564 (502.92 Dalton), suggesting that part of the inhibitor molecule was lost upon binding, while the remaining part was conjugated to USP7 core presumably at a single site.

USP7 catalytic core domain contains seven cysteine residues of which four are surface exposed and could potentially be modified by reactive small molecules [33]. To identify the conjugation site(s) of P217564 on USP7, the compound-bound USP7 core was subjected to trypsin digestion followed by LC-MS/MS analysis which revealed that P217564 forms a covalent adduct on USP7 at its active site cysteine (Cys223, Fig 4C). To confirm whether the active site cysteine is the only site targeted by P217564, we utilized an active site cysteine to alanine mutant form of USP7 core (USP7 core C223A). As expected, catalytically inactive USP7 core C223A do not react with Ub-VME (Fig 4A). Mutant USP7 core protein was incubated with P217564 and analyzed by LC-MS. Remarkably, replacement of the active site cysteine with alanine completely blocked the formation of compound adduct on the USP7 mutant core (Fig 4B), confirming that the active site cysteine is the only conjugation site for P217564 under these conditions. Thus, P217564 is a relatively selective, active site-directed covalent inhibitor of USP7. The parent compound P5091 also selectively formed adduct on the active site cysteine of USP7 (S3 Fig), indicating that both compounds exhibit similar mode of covalent USP7 inhibition.

The N-terminal TRAF domain and C-terminal UBL domains of USP7 have been shown to be responsible for substrate recognition and interaction [34]. The active site-targeted mechanism of P217564 suggests that this inhibitor might not affect the interaction of USP7 with its substrates. To verify this, *in vitro* co-immunoprecipitation (Co-IP) experiment was carried out using recombinant full length USP7 and HDM2. Recombinant USP7 was pretreated with either DMSO or 10 μM P217564 for 2 hours, incubated with HDM2 and then subjected to co-immunoprecipitation. Although USP7 activity was nearly completely inhibited after 2 hours-treatment with compound as shown by the Ub-VME assay, the interaction of USP7 with HDM2 was not affected. (S4 Fig). This result indicates that the active-site targeted P217564 does not interfere with the USP7 interaction with its substrate HDM2.

### P217564 regulates the level of USP7 substrates in cells and exerts a durable inhibitory effect

**Fig 5:**
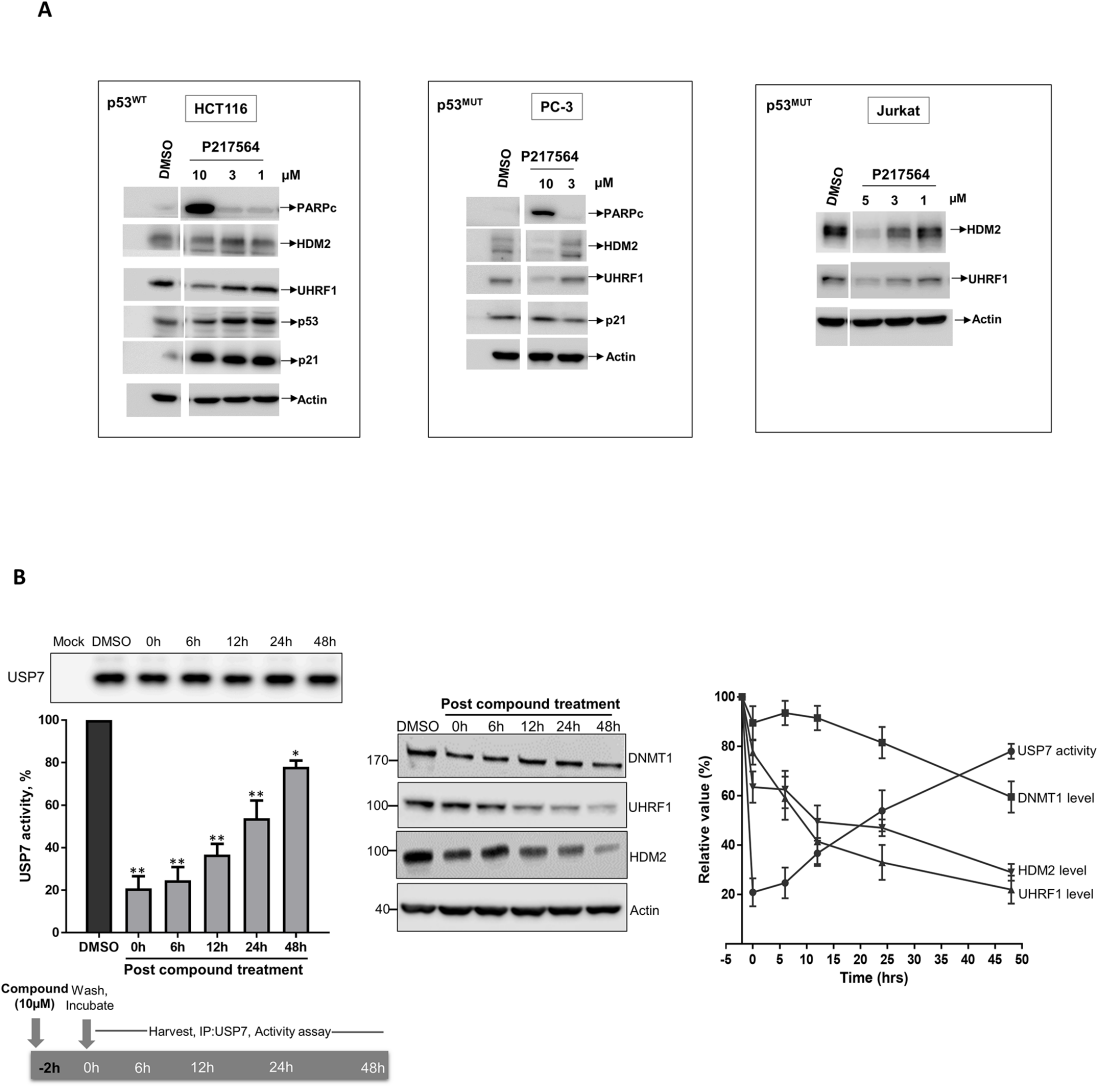
P217564 regulates the degradation of USP7 substrates and shows durable inhibition of USP7. (A) HCT116, PC-3, and Jurkat cells were treated with DMSO or P217564 at indicated concentrations for 24 hours; crude cell extracts were subjected to SDS-PAGE electrophoresis, followed by IB with indicated antibodies. (B) P217564 produces long-lasting USP7 inhibition in cells. Jurkat cells were treated with 10μM P217564 for 2 hours, the compound was washed out, and the USP7 activity was measured at various times up to 48 hours using the USP7 IP/activity assay. Cellular levels of USP7 substrates including UHRF1, DNMT1, and HDM2 were detected by Western blot. Anti-Actin blot was used as loading control.

Inhibition or knock-down of USP7 has been shown to induce apoptosis through both p53-dependent and independent pathways [21, 25]. To test the biological effect of P217564, p53 wild type (HCT116) and p53 mutant (PC-3) human cancer cell lines were incubated with P217564 for 24 hours. As shown in Fig 5A, P217564 induced apoptosis in both p53 wild type (HCT116) and mutant (PC-3) cell lines at a concentration of 10 μM, as indicated by a strong PARP (poly ADP-ribose polymerase) cleavage band, a widely used marker for apoptosis. In addition, P217564 is able to induce apoptosis in Jurkat cells, represented by a dose- and time-dependent accumulation of Annexin V and Propidium Iodide (PI) positive cells (S5 Fig). As expected, p53 protein was stabilized in HCT116 (p53^WT^) cells upon P217564 treatment, which in turn induced the expression of the cyclin-dependent kinase inhibitor p21 (Fig 5A). This result indicates that P217564 induced the functional activation of p53 in HCT116 cells. Not surprisingly, the p21 level did not change appreciably in PC-3 (p53^MUT^) cells upon compound treatment, owing to the lack of functional p53 in PC-3 cells. The transcriptional levels of MDM2, p53, and Tip60 in HCT116 cells were also analyzed after P217564-treatment. No detectable change of p53 and Tip60 mRNA level was observed after 6 and 24 hours P217564-treatment. However, MDM2 mRNA level increased significantly after compound treatment, which is consistent with previous reports that MDM2 itself is a transcriptional target of p53, and stabilization of wild type p53 after USP7 inhibition results in increase in MDM2 mRNA level (S6 Fig).

UHRF1 (Ubiquitin-like, with PHD and RING finger domains-1) and USP7 interact in cells, and USP7 is a regulator of UHRF1 stability by antagonizing ubiquitination-mediated proteasomal degradation of UHRF1 [35, 36]. Knockdown of USP7 has been shown to result in reduced UHRF1 levels in HCT116 cells [35]; we therefore tested whether P217564 treatment affected the endogenous level of UHRF1 in these cells. HCT116 cells treated with P217564 for 24 hours underwent dose-dependent downregulation of UHRF1. Similar results were observed in PC-3 and Jurkat cells, indicating that the USP7-UHRF1 regulation mechanism is conserved across cells originating from different tissues (Fig 5A).

Covalent inhibitors in general are known to have long target residence times leading to prolonged downstream biological effects. We tested the duration of the inhibitory effect of P217564 using cellular recovery as an endpoint. Jurkat cells were pulse treated with 10 μM P217564 for 2 hours, and then the free compound was washed out; cells were cultured with fresh medium and harvested at various incubation times; and USP7 was isolated by immunoprecipitation for quantification of catalytic activity using the Ub-EK_L_ based assay.

As shown in Fig 5B (Left), incubation with 10 μM compound for 2 hours led to ~80% inhibition of cellular USP7 activity, and the activity of USP7 slowly recovered post compound treatment, probably through synthesis of new USP7 proteins, revealing the long-lasting inhibitory effect of P217564. Cellular levels of USP7 substrates were also monitored during the recovery process. Interestingly, the levels of all tested USP7 substrates including HDM2, UHRF1 and DNMT1 continuously decreased while the activity of USP7 was recovering (Fig 5B), indicating that covalent irreversible inhibition of USP7 activity resulted in sustained changes in the levels of USP7 substrates.

### P217564 regulates the stability of USP7

**Fig 6:**
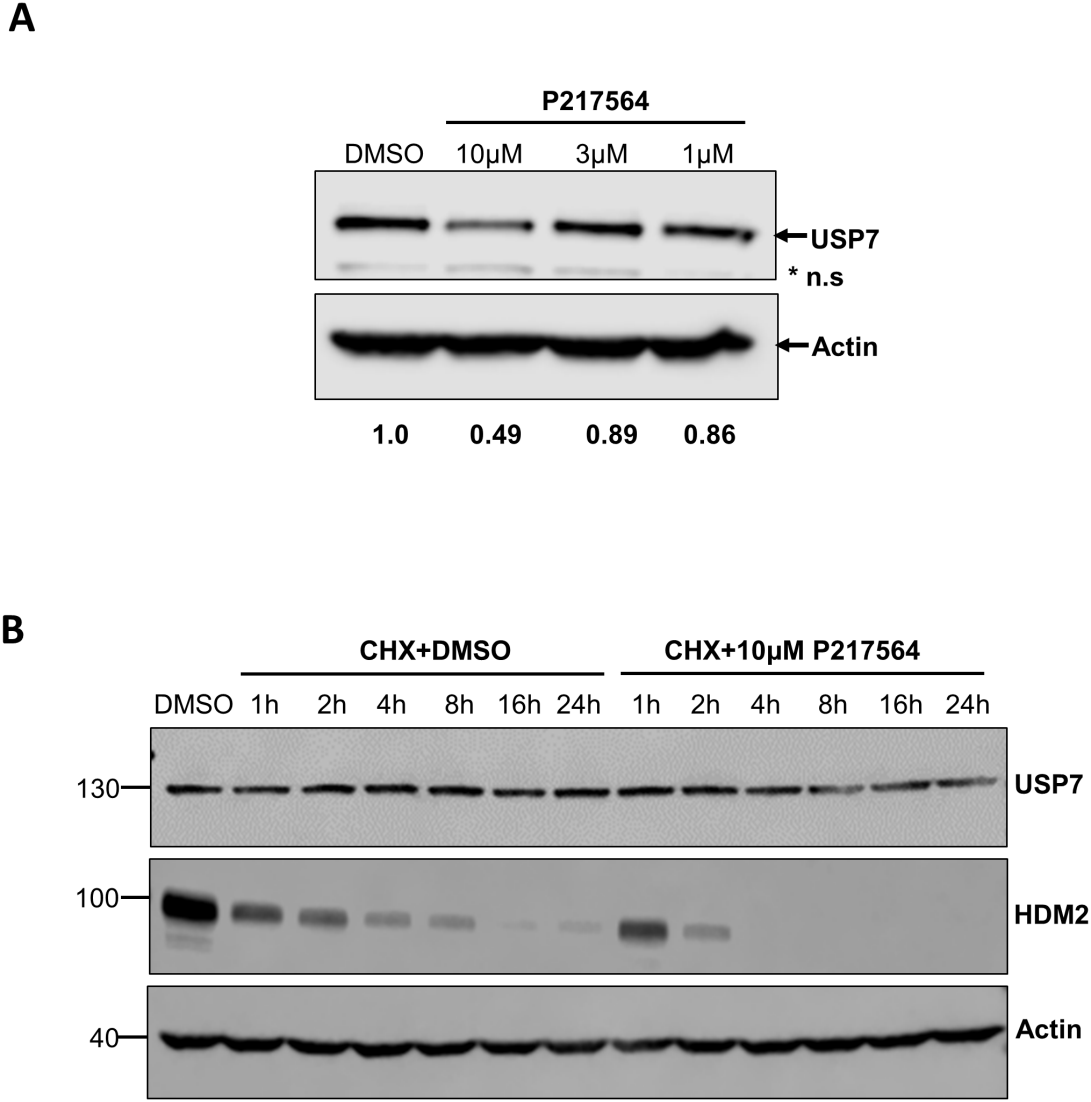
Selective USP7 inhibitor P217564 regulates the stability of USP7. (A) P217564 decreases the USP7 level in a dose-dependent manner. Jurkat cells were treated with DMSO or P217564 at indicated concentrations for 24 hours; crude cell extracts were subjected to SDS-PAGE electrophoresis, followed by IB with indicated antibodies. *n.s indicates non-specific band. Relative quantitation of band intensity is given. (B) P217564 decreases the half-life of USP7. HCT116 cells were treated with DMSO or P217564 in the presence of 50ug/mL cycloheximide (CHX), and the levels of USP7 and HDM2 were analyzed using western blotting at indicated time points. The IB of Actin was used as the loading control.

Self-regulation is a common mechanism for both E3 ligases and DUBs in regulating their own stability. Auto-ubiquitination of E3 ligases can promote their degradation whereas the enzymatic activity of certain DUBs was reported to be essential for their own stabilization [37]. USP7 has been reported to be ubiquitinated [38], which prompted us to investigate whether USP7 inhibitor P217564 affected the ubiquitylation and stability of USP7. Jurkat cells treated with P217564 for 24 hours resulted in a moderate decrease in levels of USP7 (Fig 6A), suggesting that USP7 stability is regulated by its own isopeptidase activity. In addition, the half-life of USP7 was investigated using a cycloheximide (CHX) chase assay. Interestingly, no obvious decrease of USP7 level in HCT116 cells was observed even after 24 hours of CHX treatment indicating that USP7 is a stable protein. However, HCT116 cells simultaneously treated with CHX and P217564 resulted in a decrease in USP7 levels (Fig 6B). Under the same conditions, the level of HDM2, a previously reported USP7 substrate gradually decreased upon CHX treatment, and the degradation was accelerated in the presence of P217564 (Fig 6B). These results suggested that USP7 is a very stable protein (half-life > 24 hours) and its stability is regulated either by its own isopeptidase activity or by other DUB(s) that are inhibited by P217564.

### Ubiquitination level and chain topology of USP7 substrates in cells after treatment with P217564

USP7 regulates the stability of its substrates by removing ubiquitin from them, preventing their degradation in the proteasome. Inhibition of USP7 is predicted to lead to a transient increase of the ubiquitination level of USP7 substrates. To evaluate further the cellular efficacy of P217564 in targeting USP7, we tested the ability of this compound to modulate the ubiquitination status of endogenous USP7 substrates. Jurkat cells were incubated with or without P217564 in the presence or absence of the proteasome inhibitor bortezomib (BTZ) for 2 hours, and total ubiquitinated proteins were then isolated from cell extracts using TUBE pull down [39]. Total pull down products were subjected to SDS-PAGE electrophoresis, transferred to PVDF membrane, and then immunoblotted with antibodies against several known USP7 substrates as well as ubiquitin. Although total ubiquitination dramatically increased when cells were co-treated with P217564 and BTZ, none of the two known USP7 substrates contained an increased level of polyubiquitination (S7 Fig). Detection of the ubiquitination level of endogenous substrates using this traditional immunoprecipitation/immunoblot method has been found to be difficult and highly substrate and antibody-dependent. Possible reasons for this result are: 1) only a very small fraction of a given protein is ubiquitinated at any given time, 2) the heterogeneity of length of polyubiquitin chain leads to non-uniform migration of modified proteins on SDS-PAGE, further decreasing the ubiquitination signal of a given substrate, and 3) ubiquitination may influence substrate recognition by the various antibodies.

**Fig 7:**
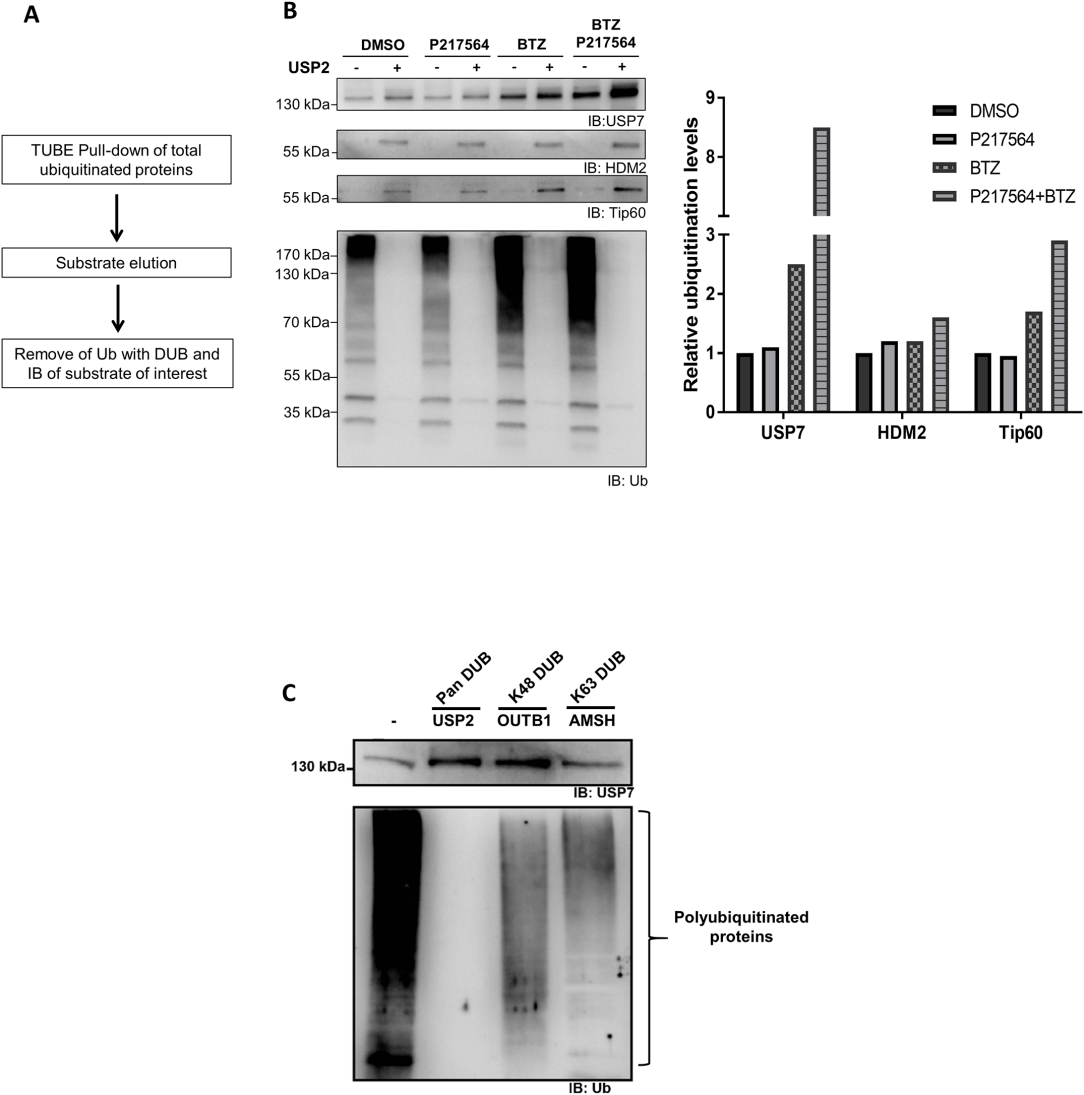
DUB-based UbiTest to capture P217564-induced ubiquitination of USP7 substrates and analyze their polyubiquitin chain topology. (A) Schematic representation of DUB-based UbiTest. (B) Jurkat cells were treated with or without P217564 in the presence or absence of bortezomib (BTZ) for 2 hours. Total ubiquitinated proteins were then isolated from crude cell extracts using tandem ubiquitin binding entity (TUBE). All pull-down products were eluted, digested with USP2, and then subjected to IB analysis with indicated antibodies (left). The increased signal of individual substrate post USP2 digestion represents the amount of ubiquitination of each substrate. The relative change of the ubiquitination level of each substrate post compound treatment was quantified (right). (C) Samples were digested with USP2, a broad spectrum DUB, or with K48 (OUTB1) or K63 (AMSH) chain type specific DUBs, and followed by IB analysis of substrates with indicated antibodies.

To overcome these obstacles to the detection of endogenous substrate ubiquitination, a DUB-based UbiTest method was applied (Fig 7A). The TUBE pull-down products (total ubiquitinated proteins) were eluted and digested using a broad-spectrum DUB (USP2 core) prior to the immunoblot analysis (Fig 7A). An increased signal for the protein of interest that is unmasked by USP2 treatment demonstrates that the protein was in fact ubiquitinated even if there was no clear reactivity in the untreated sample. As shown in Fig 7B, the ubiquitination levels of tested USP7 substrates all increased upon P217564 and BTZ co-treatment, confirming the ability of P217564 to block USP7-mediated de-ubiquitination of its substrates. The ubiquitination level of USP7 also increased upon P217564 and BTZ co-treatment, consistent with the potential self-regulation mechanism of USP7 described above.

The fate of ubiquitinated proteins is determined by the topology of their polyubiquitin chains. K48-linked polyubiquitin chains function as proteolytic signals and deliver substrates for proteasomal degradation, whereas K63-linked polyubiquitin chains are normally involved in non-proteolytic regulation [40]. To decipher the ubiquitin chain topology of USP7 substrate after P217564 treatment, TUBE pull-down products from compound-treated Jurkat cells were eluted and digested with the nonselective USP2 or with K48- or K63- specific DUBs, and then subjected to immunoblotting with antibodies against USP7. As shown in Fig 7C, although the K63 specific DUB (AMSH) could largely decrease the total ubiquitination signal, no detectable increase of USP7 signal was observed. Interestingly, the increase of USP7 signal after K48 specific DUB treatment is roughly equal to the increase of signal after USP2 core treatment, indicating that USP7 is predominantly modified by K48-linked polyubiquitin chain upon P217564 treatment, consistent with its increased proteasomal degradation after P217564 treatment.

### Effects of P217564 in on the USP7 activity of Foxp3+ Tregs and their suppressive function

**Fig 8:**
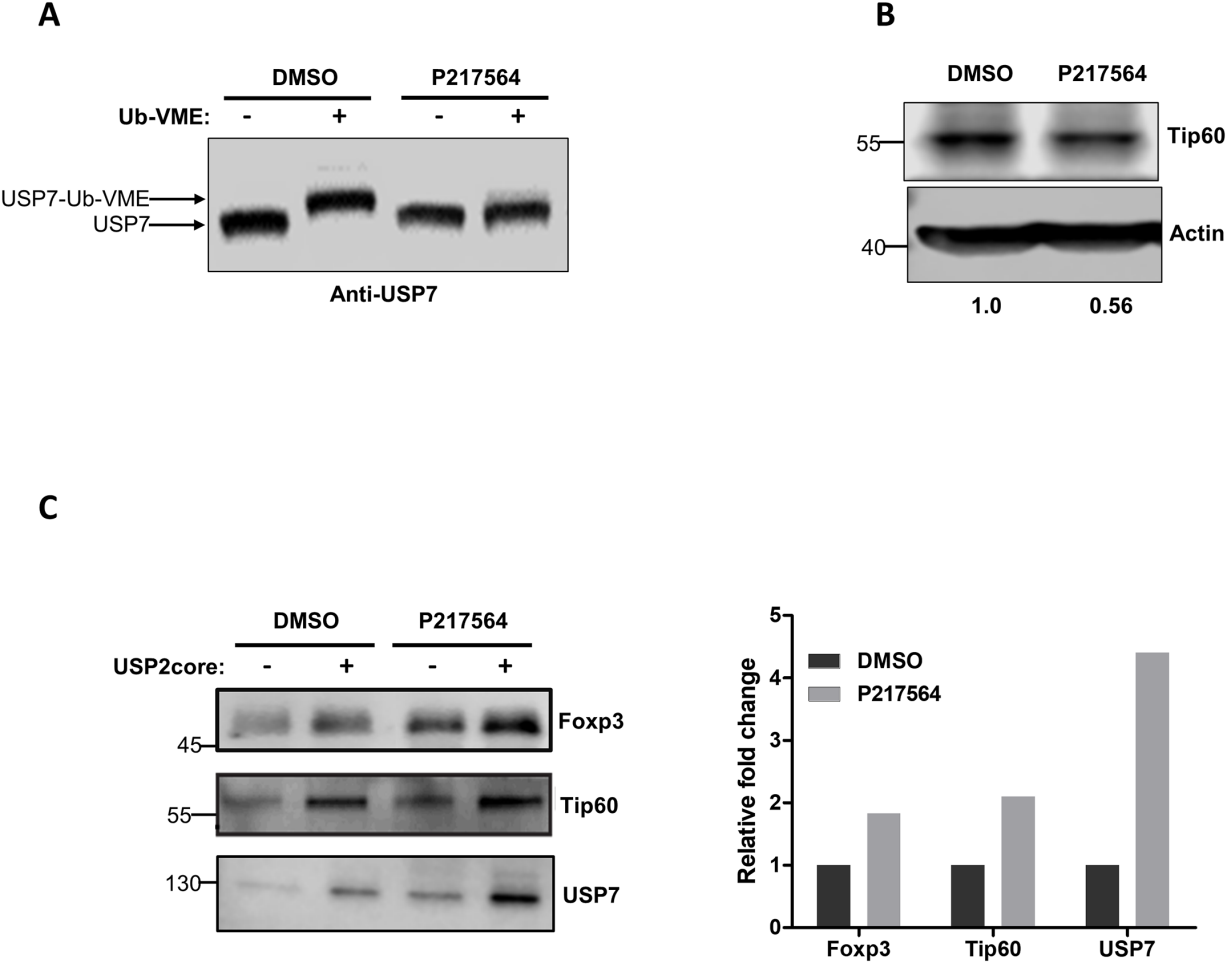
P217564 inactivates USP7, resulting in increased ubiquitination and degradation of Tip60 and Foxp3 in Treg cells. (A) Treg cells were isolated from mice and treated with 10 μM P217564 for 2 hours; crude cell extracts were incubated with Ub-VME probe, followed by IB against USP7 to analyze the formation of USP7-Ub-VME complexes. (B) Mouse Treg cells were treated with DMSO or 10 μM P217564 for 2 hours; crude cell extracts were subjected to SDS-PAGE electrophoresis, followed by IB with indicated antibodies. Relative quantitation of band intensity is shown. (C) P217564-induced ubiquitination of Tip60, Foxp3 and USP7 in Treg cells was analyzed using the DUB-based UbiTest described in Fig 6.

Stable expression of functional Foxp3 is essential for the development and maintenance of immunosuppressive functions of Treg cells, and the histone/protein acetyltransferase, Tip60, plays a dominant role in promoting Foxp3 function and stability in Treg cells [17]. Recent studies revealed that USP7 is essential for Treg cell functions primarily by stabilizing both Foxp3 and Tip60. Therefore, pharmacological inhibition of USP7 is a promising strategy for impairing Treg cell function. To test the ability of our selective USP7 inhibitor P217564 to impair Treg function, mouse Treg cells were isolated and treated with 10 μM P217564 for 2 hours. Results from the Ub-VME-based assay showed that the activity of USP7 in Treg cells was inhibited nearly completely upon P217564 treatment (Fig 8A). The level of Tip60 protein also decreased ~50% upon 2 hours of compound treatment (Fig 8B) and analysis of mRNA level under similar treatment conditions revealed that Tip60 mRNA was also decreased (S8 Fig) in Tregs. Moreover, P217564 treatment also increased the level of ubiquitination of USP7 substrates in Tregs, including both Foxp3 and Tip60 (Fig 8C), indicating that P217564 could alter functions of Foxp3 and Tip60 by promoting their ubiquitination.

**Fig 9:**
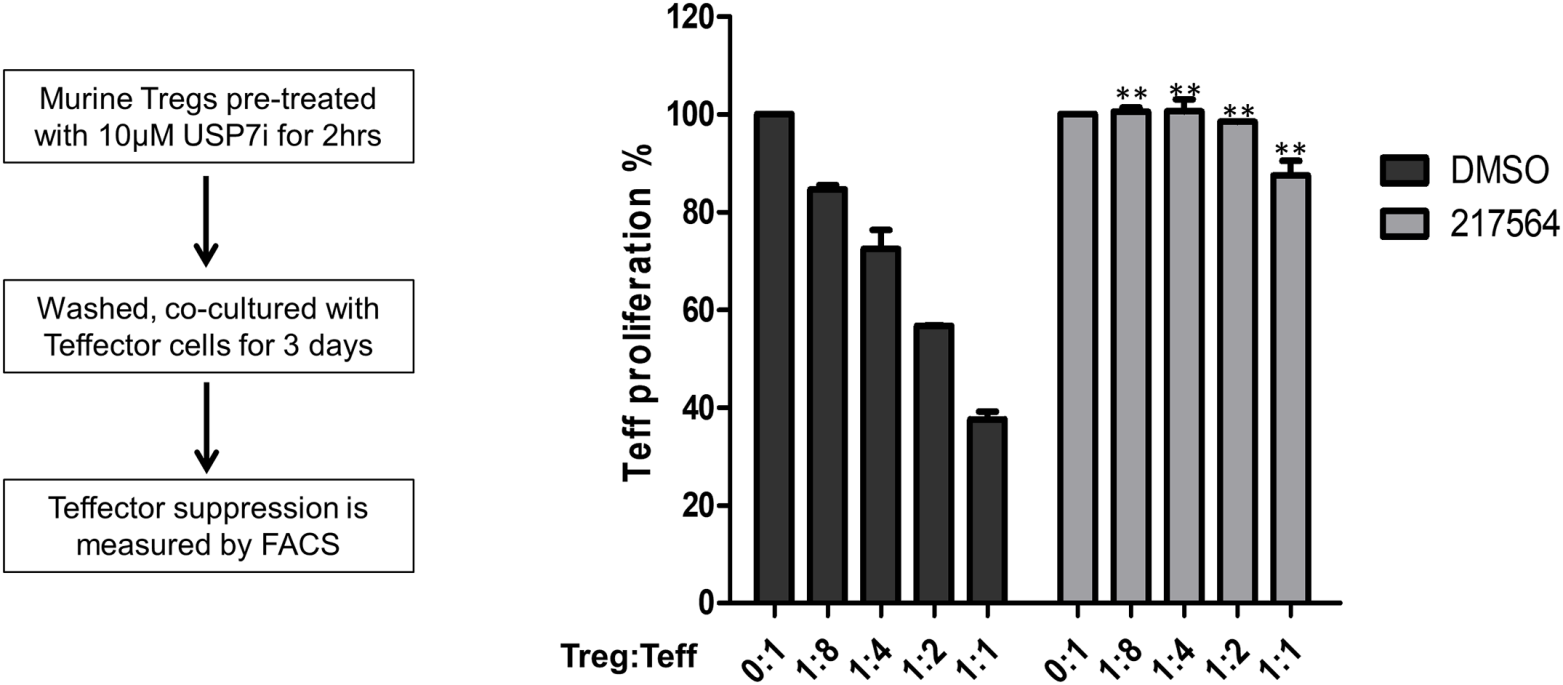
P217564 impairs suppressive function of Treg cells *ex vivo*. Murine Foxp3+ Treg cells were pre-incubated with DMSO or 10 μM P217564 for 2 hours, washed and co-cultured with T-effector cells for 3 days, at ratios of Treg to Teff cells ranging from 0:1 to 1:1. The proliferation of T-effector cells under the various conditions was analyzed by flow cytometry.

To test the ability of P217564 to impair Treg cell function, mouse Foxp3+ Treg cells were pulse treated with 10 μM P217564 for 2 hours, in which condition USP7 was nearly completely inhibited (Fig 8A), washed free of compound, and then co-cultured with T-effector cells at various ratios for 3 days. Residual T-effector cell proliferation was then analyzed by flow cytometry. Remarkably, the suppressive function of Treg cells was almost completely abrogated (Fig 9), and these sustained inhibitory effects on Treg cell function correlated well with durable USP7 inhibition efficacy by the covalent USP7 inhibitor P217564.

## Discussion

In this study, we investigated the mechanism of action of P217564 an improved USP7 inhibitor with known anticancer activity—through its ability to impair Treg function. We have shown that the P217564 inhibits Treg USP7 activity and destabilizes the Treg cell proteins, Tip60. Tip60 plays a unique role in promoting Foxp3 acetylation and dimerization, which are required for Foxp3 to interact with DNA and regulate gene transcription in Treg cells [17]. Both USP7 and Tip60 are widely expressed in various cells types. Since Foxp3 is exquisitely specific for Treg cells, and the effect of USP7 and Tip60 on Treg function is exerted by modulating Foxp3 stability and function [20], inhibition of USP7 represents a relatively selective means of targeting Treg function, since it had little or no effect on T-effector cells. The pharmacological consequences of USP7 inhibition in the immune setting derive in large part from the dependence of Treg cells on Foxp3 (and on its regulator Tip60) for activation and the ability of Tregs to restrain Teffector cells and thereby promote immune evasion. Our results constitute proof of concept that Treg function can be attenuated through pharmacological inhibition of USP7 with small molecule inhibitors.

USP7 has been shown to regulate the stability of numerous proteins, in addition to Tip60 and Foxp3, which are broadly characterized as oncogenesis-related proteins. For instance, USP7 plays a key role in regulating the ubiquitination and stability of the RING-finger E3 ligase HDM2, which in turn controls the stability of the tumor suppressor p53 [41–43]. In addition to its well characterized role in regulating HDM2 and p53 levels, USP7 modulates the stability of a number of additional proteins such as FOXO4, PTEN, claspin and UHRF1 [36, 44–47]; thus, inhibiting USP7 has the potential for direct anti-tumor activity against both p53 wild type and p53 mutant tumors [48]. In support of this notion, USP7 selective inhibitors have been found to significantly repress the growth of multiple tumor xenograft models in immunodeficient mice, including multiple myeloma, B cell leukemia, and neuroblastoma; in these studies, decreased tumor volume translated into increased survival of the tumor-implanted mice [21, 24, 49]. Hence, inhibition of USP7 may provide dual anti-tumor activities—direct tumoricidal effects and immune-mediated tumor elimination. The relative contributions of these two distinct mechanisms could be tumor-type dependent. In tumors that largely rely on up-regulated USP7 activity for survival and growth, the direct anti-tumor (cytotoxic) effect might be dominant; for tumors that depend on Treg cells for immune evasion, USP7 inhibitor mediated Treg suppression may prevail. In patients, tumors that rely on both mechanisms may be the best candidates for treatment with USP7 inhibitors. The dose of USP7 inhibitor could also be critical. Nearly complete inhibition of enzyme activity is usually required for direct anti-tumor effect, but partial inhibition of USP7 may be enough to tilt the balance of immune homeostasis and initiate immune attack on tumor cells.

In addition, we have demonstrated the covalent interaction between the USP7 active site and the selective USP7 inhibitor P217564, which explains its durable inhibitory efficacy. Based on this covalent mechanism, we developed assays to evaluate the compound occupancy of USP7, which itself can be used as the pharmacodynamic (PD) marker. Several USP7 substrates including Tip60, UHRF1, DNMT1 and HDM2 are downregulated in response to USP7 inhibition. Interestingly in Treg cells, Tip60 is downregulated at the level of both protein production and stability after USP7 inhibitor treatment. Previous studies using cancer cell lines HCT116 and U2OS showed that knocking down USP7 did not change Tip60 mRNA levels [18]. In agreement with this, we also demonstrate that treatment with USP7 inhibitor P217564 did not result in significant changes in Tip60 mRNA levels in HCT116 cells. Thus, USP7 appears to play cell-type specific role in Tip60 regulation. Interestingly, deubiquitinases have been implicated in mRNA stability as well as biogenesis [50–53]. Given the diverse cellular functions of USP7 substrates, they could be used as specific PD markers for USP7 inhibition in *in vivo* studies. However, the dynamic nature of protein ubiquitination and tissue-specific transcriptional regulation of substrates could pose challenges in establishing robust *in vivo* biomarkers for USP7 inhibition. The covalent mechanism-based assays can be utilized for further preclinical and clinical evaluation of compound-target engagement and the relationship of this engagement with downstream efficacy. Moreover, a compound-based probe (compound with an affinity tag or a detection group) could be developed for more comprehensive compound selectivity studies, with the potential to identify the possible off-targets of the compound and to serve as a useful tool for the future *in vivo* pharmacokinetics (PK) and clinical correlate studies.

## Supporting information

S1 Fig P217564 and its parent compound P5091 inhibit USP7 and USP47 in cells. (A) P5091 inhibits USP7 in cultured cells. Jurkat cells were treated with DMSO or P5091; cell lysates were incubated with the Ub-VME probe and then immunoblotted against USP7 to determine the formation of USP7-Ub-VME complexes. (B) P217564 inhibits USP7 and USP47 in cells. Jurkat cells were treated with DMSO or P5217564; cell lysates were incubated with the Ub-VME probe and then immunoblotted against USP7 and USP47 to detect the formation of USP7-Ub-VME and USP47-Ub-VME complexes.
(TIF)

S2 Fig P217564 induces chemical shift changes in NMR spectrum of USP7 core. Overlay of ^15^N-^1^H HSQC spectra of free USP7core (blue) and USP7 core-P217564 complex (red). A close-up of a select spectral region is shown in the inset. Both free and P217564-bound NMR samples contain 5% DMSO.
(TIF)

S3 Fig P5091 binds to USP7 covalently at its active site cysteine. Purified USP7core WT or C223A was incubated with either DMSO or P5091, and then subjected to LC-MS analysis to detect the formation of compound adduct on the USP7 core protein.
(TIF)

S4 Fig P217564 does not interfere with USP7 and substrate interaction. *In vitro* Co-IP assay was performed to test the effect of P217564 on USP7-HDM2 interaction. The Co-IP of HDM2 by USP7 was not affected (S4A Fig), even though USP7 catalytic activity was nearly completely inhibited by P217564 treatment (S4B Fig).
(TIF)

S5 Fig P217564 induces dose- and time-dependent apoptosis of Jurkat cells. Jurkat cells were treated with DMSO, 1 or 5 μM P217564 for 4 or 16 hours, stained with FITC Annexin V and / Propidium Iodide (PI), and subjected to flow cytometry analysis.
(TIF)

S6 Fig Transcriptional level of USP7 substrates after P217564 treatment. HCT116 cells were treated with DMSO or 10 μM P217564 for either 6 or 24 hours. mRNAs were isolated, reverse transcribed to cDNAs, and analyzed by quantitative real-time PCR.
(TIF)

S7 Fig Difficulties inherent in the use of traditional methodology to capture and quantify P217564-induced ubiquitination of USP7 substrates. Jurkat cells were incubated with or without P217564 in the presence or absence of proteasome inhibitor bortezomib (BTZ) for 2 hours, total ubiquitinated proteins were then isolated from crude cell extracts using TUBE pull down. Total pull down products were subjected to SDS-PAGE electrophoresis, transferred to PVDF membranes, and then immunoblotted with indicated antibodies against USP7 substrates as well as total ubiquitination.
(TIF)

S8 Fig Transcriptional level of Foxp3 and Tip60 in Treg cells after P217564 treatment. Treg cells were treated with DMSO or 10 μM P217564 for 2 hours. mRNAs were isolated, reverse transcribed to cDNAs, and analyzed by quantitative real-time PCR.
(TIF)

S1 File Chemical shift perturbations in NMR spectrum of USP7 core induced by P217564. (XLSX)
