# Supplementary figures and images for: Active site-targeted covalent irreversible inhibitors of USP7 impair the functions of Foxp3+ T-regulatory cells by promoting ubiquitination of Tip60
Source: PLoS One. 2017 Dec 13;12(12):e0189744. doi: 10.1371/journal.pone.0189744 (PMC5728538; doi:10.1371/journal.pone.0189744)

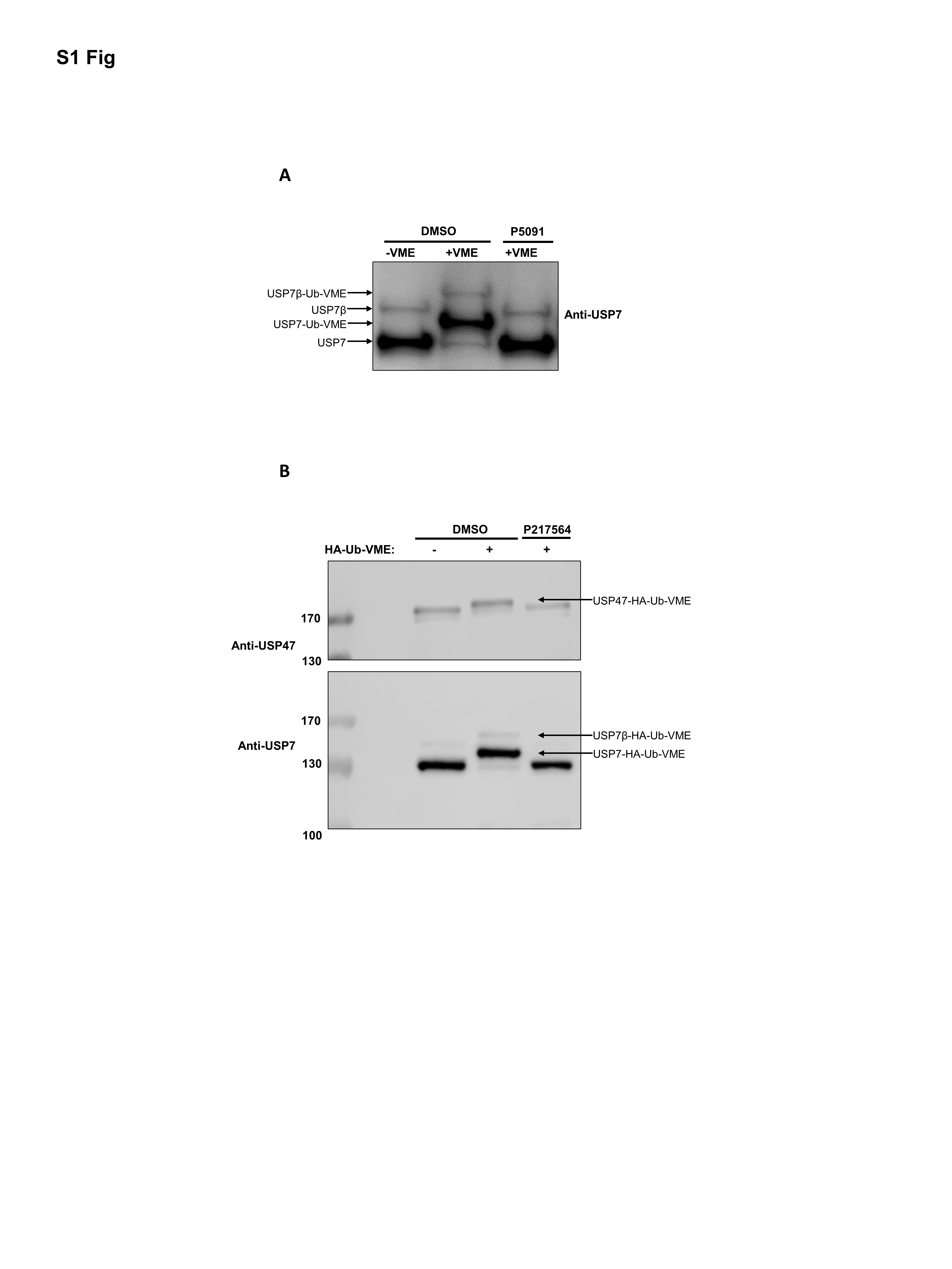

Supplement: S1 Fig — (A) P5091 inhibits USP7 in cultured cells. Jurkat cells were treated with DMSO or P5091; cell lysates were incubated with the Ub-VME probe and then immunoblotted against USP7 to determine the formation of USP7-Ub-VME complexes. (B) P217564 inhibits USP7 and USP47 in cells. Jurkat cells were treated with DMSO or P5217564; cell lysates were incubated with the Ub-VME probe and then immunoblotted against USP7 and USP47 to detect the formation of USP7-Ub-VME and USP47-Ub-VME complexes. (TIF) [file pone.0189744.s001.tif]

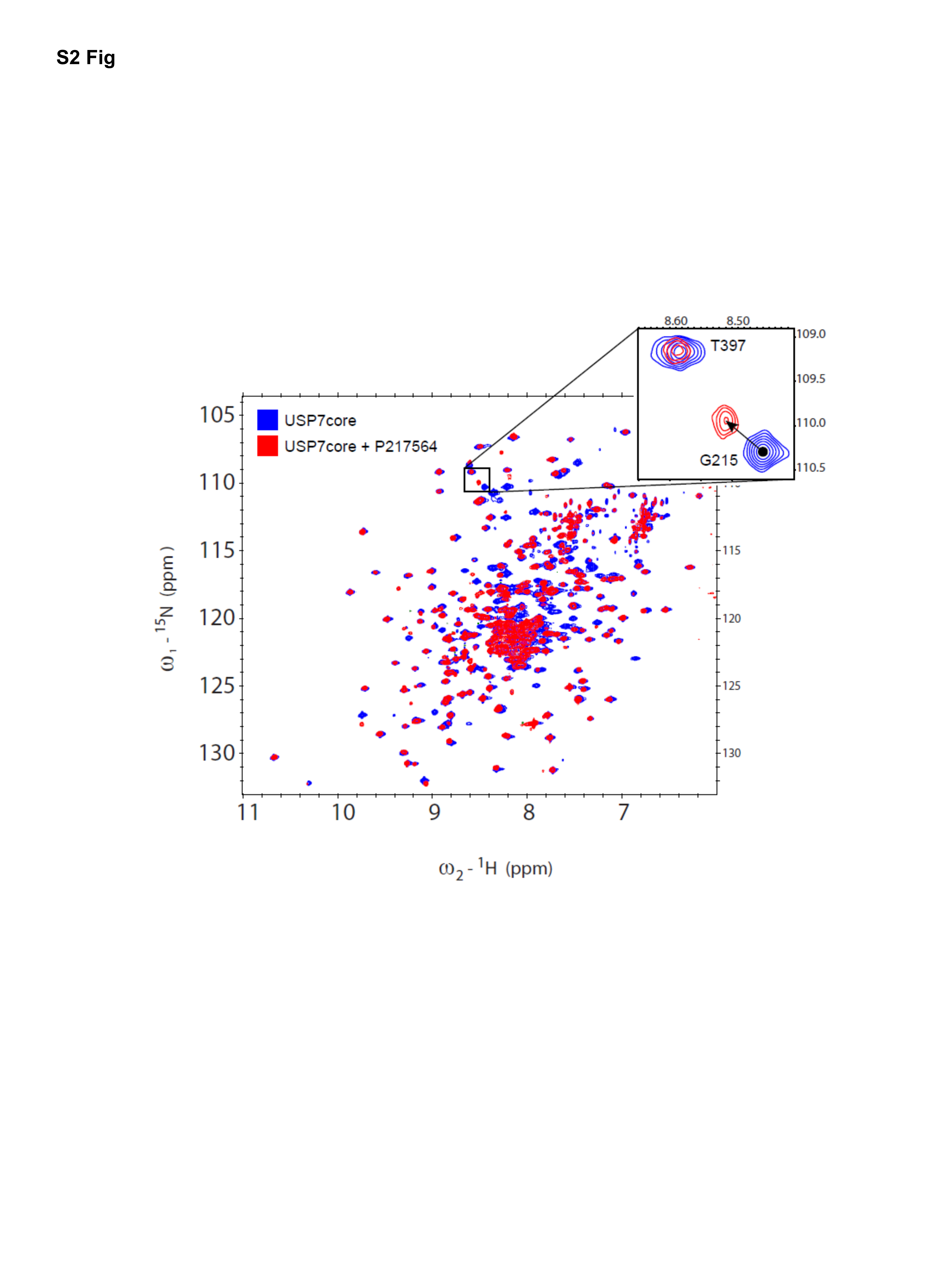

Supplement: S2 Fig — Overlay of 15N-1H HSQC spectra of free USP7core (blue) and USP7 core-P217564 complex (red). A close-up of a select spectral region is shown in the inset. Both free and P217564-bound NMR samples contain 5% DMSO. (TIF) [file pone.0189744.s002.tif]

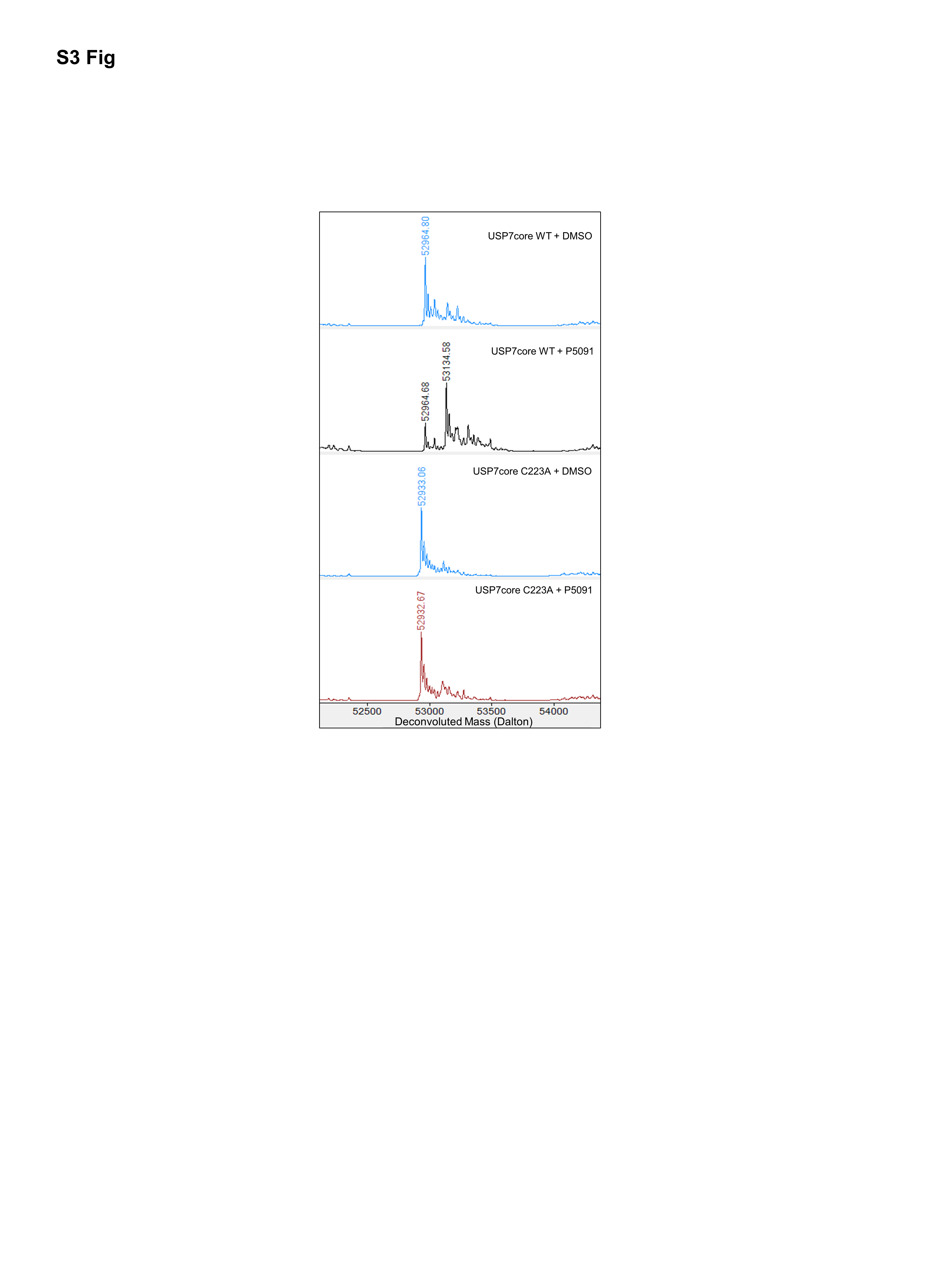

Supplement: S3 Fig — Purified USP7core WT or C223A was incubated with either DMSO or P5091, and then subjected to LC-MS analysis to detect the formation of compound adduct on the USP7 core protein. (TIF) [file pone.0189744.s003.tif]

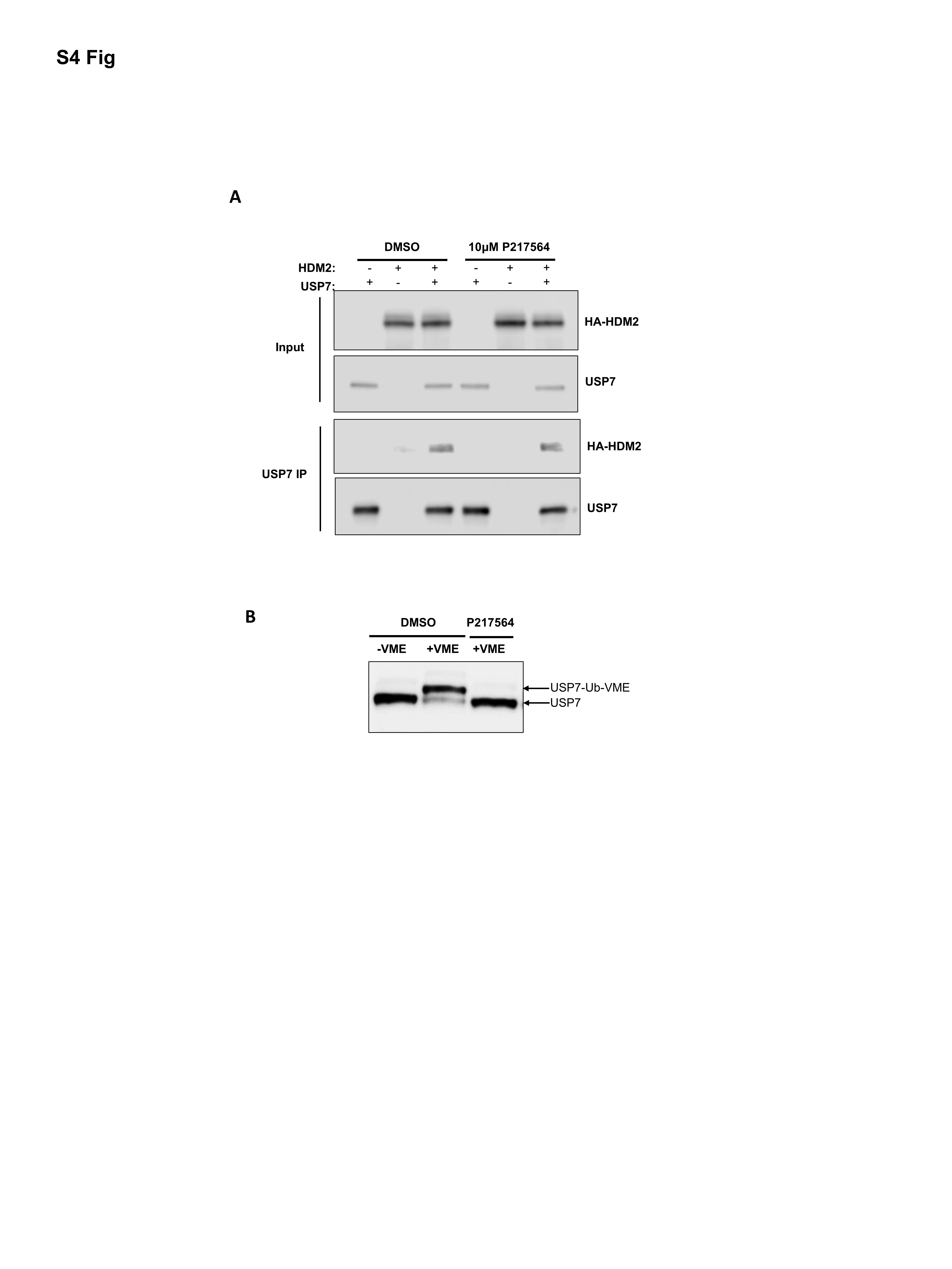

Supplement: S4 Fig — In vitro Co-IP assay was performed to test the effect of P217564 on USP7-HDM2 interaction. The Co-IP of HDM2 by USP7 was not affected (S4A Fig), even though USP7 catalytic activity was nearly completely inhibited by P217564 treatment (S4B Fig). (TIF) [file pone.0189744.s004.tif]

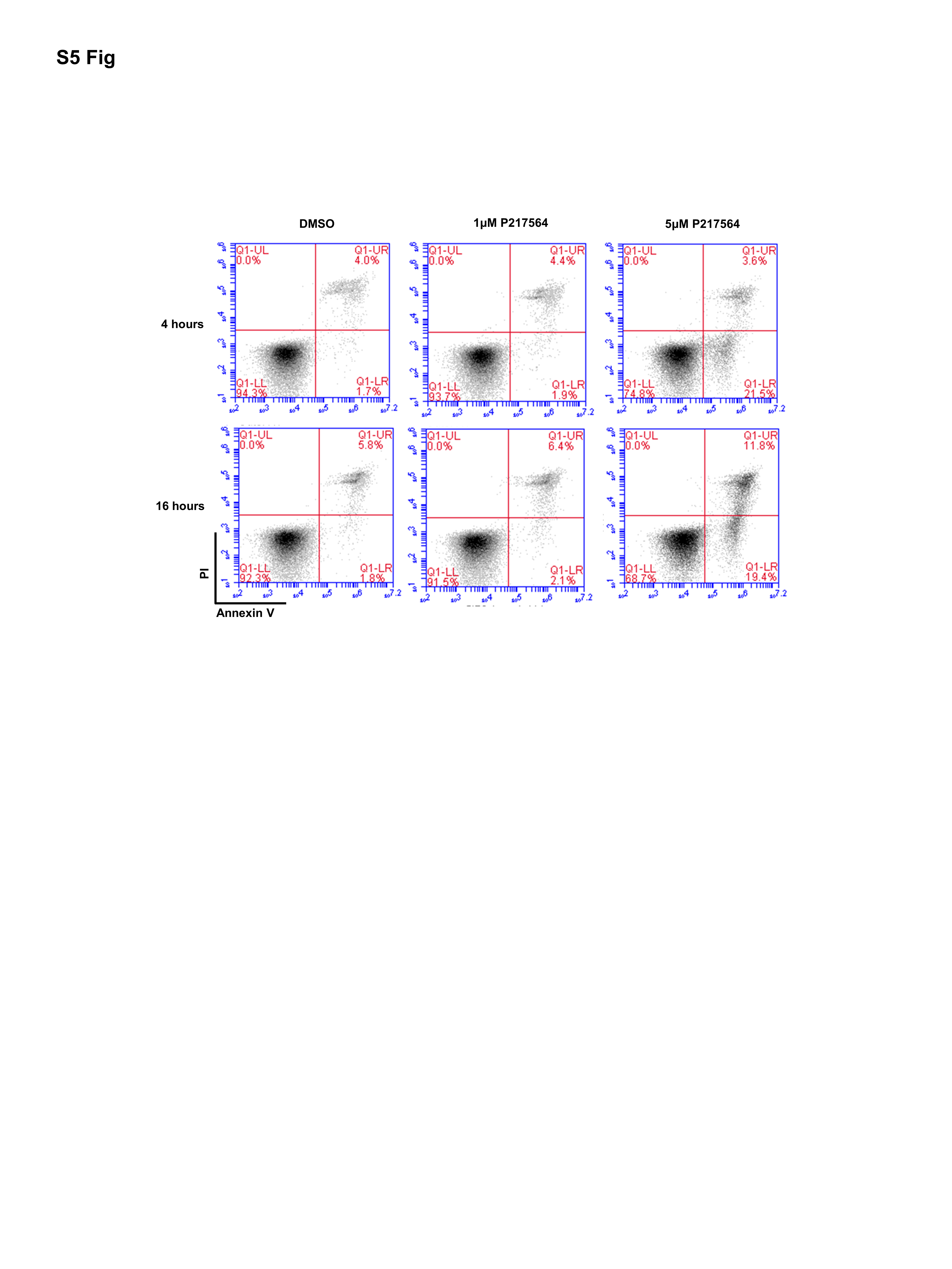

Supplement: S5 Fig — Jurkat cells were treated with DMSO, 1 or 5 μM P217564 for 4 or 16 hours, stained with FITC Annexin V and / Propidium Iodide (PI), and subjected to flow cytometry analysis. (TIF) [file pone.0189744.s005.tif]

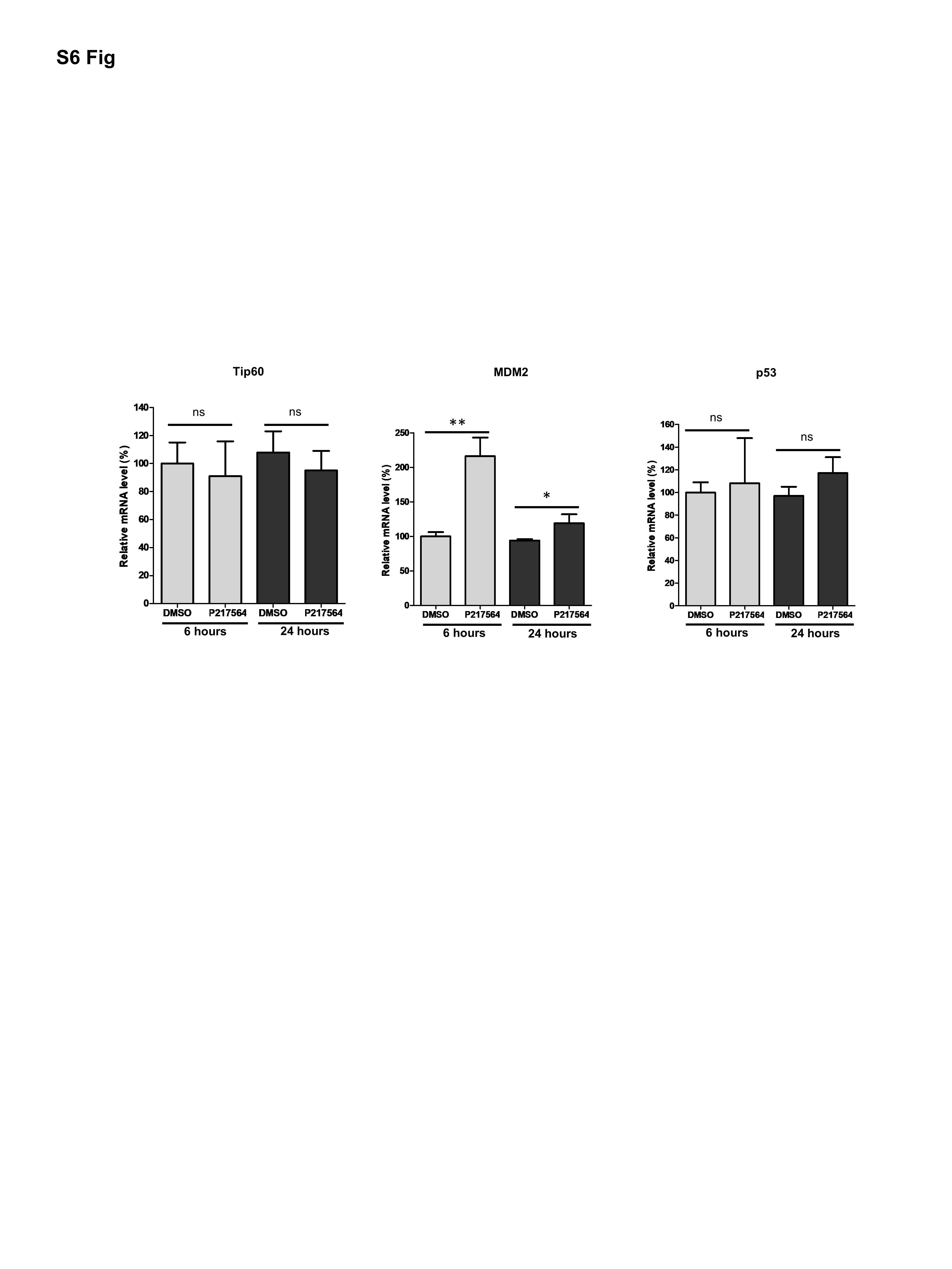

Supplement: S6 Fig — HCT116 cells were treated with DMSO or 10 μM P217564 for either 6 or 24 hours. mRNAs were isolated, reverse transcribed to cDNAs, and analyzed by quantitative real-time PCR. (TIF) [file pone.0189744.s006.tif]

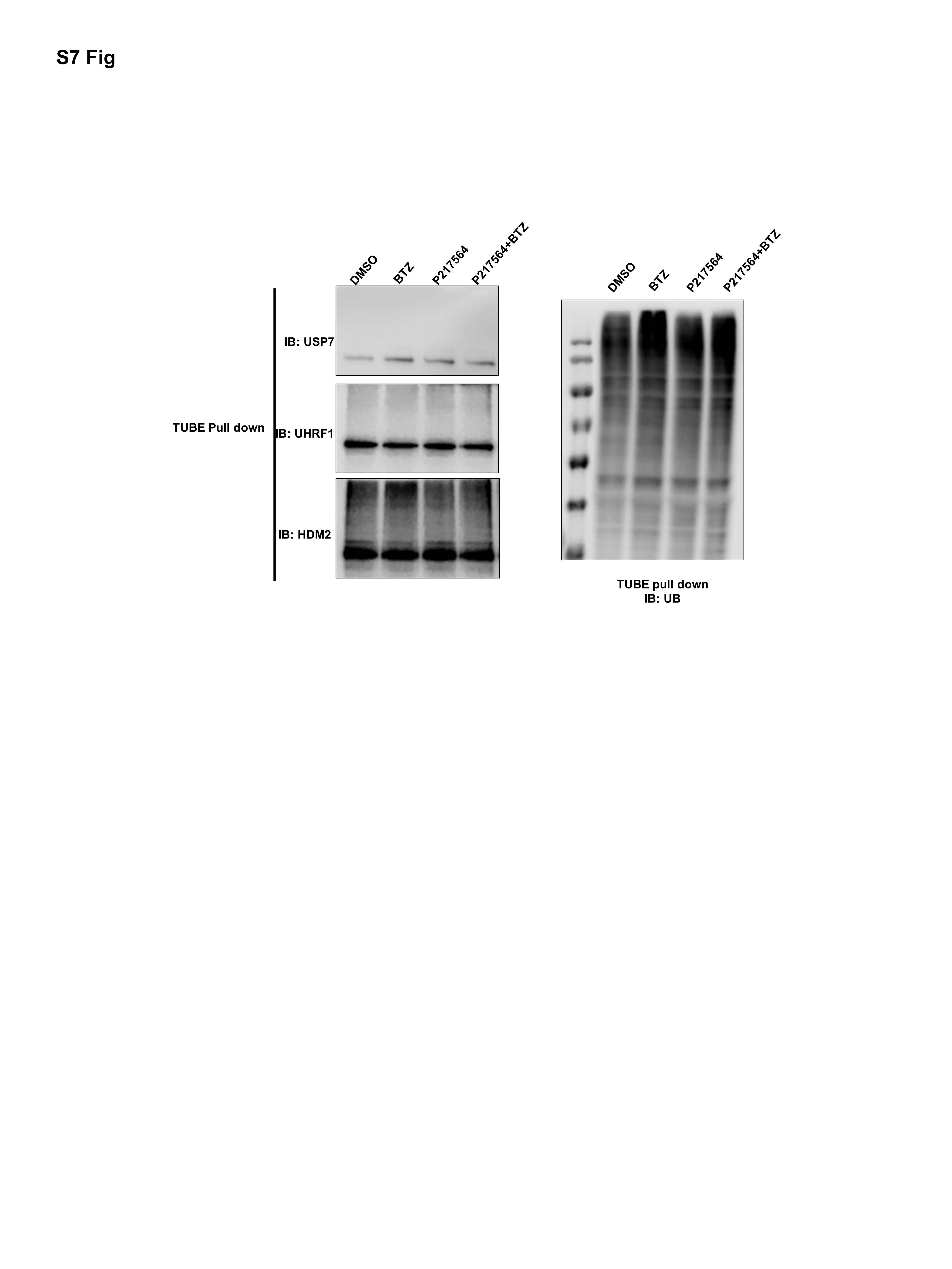

Supplement: S7 Fig — Jurkat cells were incubated with or without P217564 in the presence or absence of proteasome inhibitor bortezomib (BTZ) for 2 hours, total ubiquitinated proteins were then isolated from crude cell extracts using TUBE pull down. Total pull down products were subjected to SDS-PAGE electrophoresis, transferred to PVDF membranes, and then immunoblotted with indicated antibodies against USP7 substrates as well as total ubiquitination. (TIF) [file pone.0189744.s007.tif]

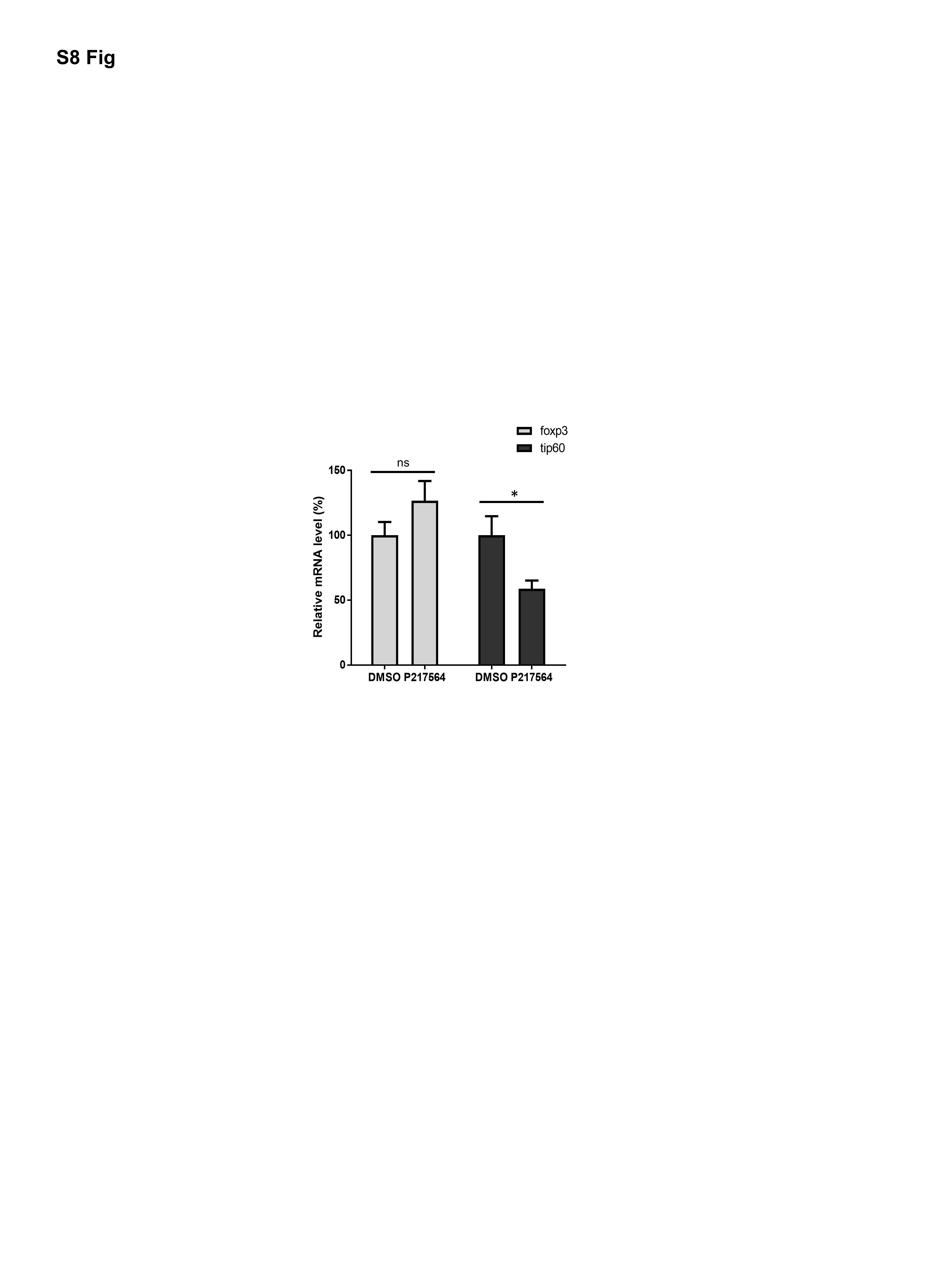

Supplement: S8 Fig — Treg cells were treated with DMSO or 10 μM P217564 for 2 hours. mRNAs were isolated, reverse transcribed to cDNAs, and analyzed by quantitative real-time PCR. (TIF) [file pone.0189744.s008.tif]
